# Warming, but Not Acidification, Restructures Epibacterial Communities of the Baltic Macroalga *Fucus vesiculosus* With Seasonal Variability

**DOI:** 10.3389/fmicb.2020.01471

**Published:** 2020-06-26

**Authors:** Birte Mensch, Sven C. Neulinger, Sven Künzel, Martin Wahl, Ruth A. Schmitz

**Affiliations:** ^1^Department of Biology, Institute of General Microbiology, Kiel University, Kiel, Germany; ^2^omics2view.consulting GbR, Kiel, Germany; ^3^Department of Evolutionary Genetics, Max Planck Institute for Evolutionary Biology, Plön, Germany; ^4^Marine Ecology Division, Research Unit Experimental Ecology, Benthic Ecology, GEOMAR Helmholtz Centre for Ocean Research Kiel, Kiel, Germany

**Keywords:** bacterial community structure, 16S rDNA amplicon sequencing, temperature, *p*CO_2_, benthocosm, macroalgal holobiont

## Abstract

Due to ocean acidification and global warming, surface seawater of the western Baltic Sea is expected to reach an average of ∼1100 μatm *p*CO_2_ and an increase of ∼5°C by the year 2100. In four consecutive experiments (spanning 10–11 weeks each) in all seasons within 1 year, the abiotic factors temperature (+5°C above *in situ*) and *p*CO_2_ (adjusted to ∼1100 μatm) were tested for their single and combined effects on epibacterial communities of the brown macroalga *Fucus vesiculosus* and on bacteria present in the surrounding seawater. The experiments were set up in three biological replicates using the Kiel Outdoor Benthocosm facility (Kiel, Germany). Phylogenetic analyses of the respective microbiota were performed by bacterial 16S (V1-V2) rDNA Illumina MiSeq amplicon sequencing after 0, 4, 8, and 10/11 weeks per season. The results demonstrate (**I**) that the bacterial community composition varied in time and (**II**) that relationships between operational taxonomic units (OTUs) within an OTU association network were mainly governed by the habitat. (**III**) Neither single *p*CO_2_ nor *p*CO_2_:Temperature interaction effects were statistically significant. However, significant impact of ocean warming was detected varying among seasons. (**IV**) An indicator OTU (iOTU) analysis identified several iOTUs that were strongly influenced by temperature in spring, summer, and winter. In the warming treatments of these three seasons, we observed decreasing numbers of bacteria that are commonly associated with a healthy marine microbial community and—particularly during spring and summer—an increase in potentially pathogenic and bacteria related to intensified microfouling. This might lead to severe consequences for the *F. vesiculosus* holobiont finally affecting the marine ecosystem.

## Introduction

Macroalgal hosts and their associated microorganisms, designated as holobionts (Egan et al., 2013; Saha and Weinberger, 2019) or synonymously termed as “metaorganisms” (Bosch and McFall-Ngai, 2011), need to cope with changing environmental conditions like ocean acidification and warming (Qiu et al., 2019; van der Loos et al., 2019). For the adaptation and resilience of a holobiont to an altered environment an optimized host-microbe interaction is predicted to be essential (Dittami et al., 2016). In marine environments macroalgae are important components of benthic communities and essential hosts for a multitude of associated microbes (Wiencke and Bischof, 2012). Due to secreted polysaccharides their nutrient-rich surfaces represent attractive targets for rapid colonization, either by various microbes from other near-by marine organisms (e.g., algae, animals) or from the surrounding seawater resulting in the establishment of an epibiotic biofilm (Armstrong et al., 2001; Steinberg and de Nys, 2002; Dang and Lovell, 2016). Since bacteria are the first colonizers and main components of microbial biofilms, they are often considered as the most diverse microorganisms on marine surfaces with important roles attributed to them (e.g., key metabolic pathways like carbon cycling) (Wahl et al., 2012; Singh and Reddy, 2014; Dang and Lovell, 2016). The epibacterial community and the colonized macroalgal host interact in multiple and complex ways (Goecke et al., 2010; Egan et al., 2013; Dang and Lovell, 2016). Bacteria can provide the algal host with beneficial substances. For example, marine epibacteria and extracellular polymeric substances (EPS) play important roles in the morphological development and healthy growth of the algal host (Singh and Reddy, 2014; Wichard, 2015). Epibacteria benefit from the nutrient-rich algal surface, since the macroalgae provide substances the attached bacteria require for growth and metabolism. For example, synthesized macroalgal polysaccharides can be digested by bacterial enzymes to obtain carbon and energy (Hehemann et al., 2012; Martin et al., 2015). Beside bacteria, also for other marine organisms the nutrient-rich algal surface is an attractive habitat for settlement. In this regard, the epibacterial layer may promote, hinder or selectively control further settlement of prokaryotic and eukaryotic organisms on the algal surface (Nasrolahi et al., 2012; Wahl et al., 2012). Microorganisms associated with macroalgae may exhibit strong antimicrobial and antifouling activities (Satheesh et al., 2016). On the other hand, macroalgae might be able to control their epibacterial layer to a certain degree through the use of attracting or defending substances. For example, for Baltic *Fucus vesiculosus* it was shown that thallus-associated compounds mediated bacterial surface colonization (Lachnit et al., 2009b, 2013; Saha et al., 2011), whereas surface-attached molecules inhibited bacterial settlement protecting the macroalgal surface against potential pathogens and microfouling (Weinberger, 2007; Wahl et al., 2010; Saha et al., 2012, 2017; Saha and Wahl, 2013; Saha and Weinberger, 2019). In general, bacterial cell densities on nutrient-rich macroalgal surfaces are high (Martin et al., 2014; Dang and Lovell, 2016). On Baltic *F. vesiculosus* an average bacterial cell density of 5 × 10^7^ per cm^2^ was reported by Stratil et al. (2013), suggesting highly complex interactions by direct cell-cell communication within the biofilm. The biofilm composition fundamentally depends on many biotic and abiotic conditions such as algal species, age, season and environmental parameters as reviewed by Martin et al. (2014). Previous studies reported that epibacterial communities differ between macroalgal host species and seasons (Lachnit et al., 2009a, 2011). Experimental studies by Stratil et al. (2013, 2014) further indicated a reshaping of native epibacterial communities on Baltic *F. vesiculosus* along temperature and salinity gradients over time.

In the context of climate change, ocean acidification and global warming are key processes driven by increasing atmospheric CO_2_ concentrations that affect marine ecosystems worldwide. It is likely that the partial pressure of CO_2_ (*p*CO_2_) will continue to increase over the next ∼100 years causing a decrease in mean pH of the oceans by approximately 0.2–0.3 pH units and global temperature is expected to increase by approximately 3–7°C (Omstedt et al., 2012; IPCC, 2013; Elken et al., 2015) with regional variability. The temperature of coastal shallow waters of the western Baltic Sea is expected to increase by up to 5°C until the year 2100 (Gräwe et al., 2013; Elken et al., 2015; Wahl et al., 2015a). Furthermore, the pH in this region is expected to decrease in average by ∼0.2 units driven by elevated *p*CO_2_ reaching an average of ∼1100 μatm (Wahl et al., 2015a). The majority of ocean acidification studies on marine microbes mainly focused on planktonic microbial communities with rather variable and inconsistent outcomes regarding effects on abundance, diversity and community composition. The results reach from changes in planktonic bacterial community composition and function (Beman et al., 2011; Kitidis et al., 2011; Krause et al., 2012; Lomas et al., 2012; Bergen et al., 2016) to mostly stable community compositions without functional changes (Newbold et al., 2012; Lindh et al., 2013; Roy et al., 2013; Oliver et al., 2014) under acidification. Incubation experiments with microbial biofilms and marine sediments resulted either in rapid responses of microbial communities to ocean acidification (Witt et al., 2011; Laverock et al., 2013; Braeckman et al., 2014; Tait et al., 2014), or in minor effects of acidification on community composition and function (Tait et al., 2013; Gazeau et al., 2014). Currently, the response of epibacteria on algae to ocean acidification is scarce. In particular, the response of epibacterial communities on marine macroalgae exposed to combined acidification and warming under near-natural conditions including seasonal and daily fluctuations in seawater parameters, remains unexplored. One exception is our recent study Mensch et al. (2016), that revealed only minor *p*CO_2_ but strong temperature effects on the bacterial communities attached to the Wadden Sea *F. vesiculosus* forma *mytili* (*F. mytili*) and in surrounding seawater. This study was performed in the outdoor tidal benthic mesocosms on the island of Sylt, Germany (Pansch et al., 2016), using a similar experimental design as outlined in the current study, however, was limited to one spring experiment (Mensch et al., 2016).

In most of the Baltic Sea, a key habitat-forming species is the perennial brown macroalga *F. vesiculosus* of the class *Fucophyceae* (*Phaeophyceae*) (Torn et al., 2006; Rönnbäck et al., 2007). *F. vesiculosus* individuals grow on hard substrata, in the western Baltic Sea typically down to a water depth of 2 m (Kautsky et al., 1986; Vogt and Schramm, 1991). Their thalli possess gas vesicles favoring an upright habitus and, thus enhancing light harvest. Together with its associated micro- and macro-organisms, *F. vesiculosus* represents a benthic community of high ecological importance for the coastal shallow waters of the western Baltic Sea. Thus, it was of special interest to investigate the impacts of future ocean acidification and warming events on the *F. vesiculosus* holobiont under near-natural experimental conditions across all seasons within 1 year. In this context, the use of mesocosms has become a popular tool in climate change research (Stewart et al., 2013).

Our experiments were conducted in the Kiel Outdoor Benthocosms (KOB) described by Wahl et al. (2015a), followed by phylogenetic analyses by Illumina amplicon sequencing of bacterial 16S rRNA genes. The study addressed the following questions. (**I**) What is the composition of the bacterial communities on the surface of *F. vesiculosus* and within the surrounding seawater and how do they restructure throughout all four seasons? (**II**) Which operational taxonomic units (OTUs) are strongly correlated among the OTUs that were present across the majority (≥60%) of all samples? (**III**) Do ocean acidification and warming impact the bacterial communities and will this impact vary among seasons? And finally, (**IV**) which bacterial taxa suffer or benefit from warming, identified by an indicator OTU (iOTU) analysis (Fortunato et al., 2013)?

## Materials and Methods

### The Kiel Outdoor Benthocosms

The benthocosms were constructed to simulate near-natural Baltic Sea shallow underwater climate scenarios (Supplementary Figure S1). For a detailed description of the KOB facility (located at the Kiel Fjord, Kiel, Germany), the experimental design and the underlying water parameters of the performed seasonal experiments see Wahl et al. (2015a). Briefly, the floating outdoor facility consists of six tanks with two independent subunits resulting in 12 experimental units with a seawater capacity of 1500 L each. Seawater was constantly supplied from the Kiel Fjord via a pipeline located at 1 m depth (with flow-through of ∼1.3 times the tank volume per 24 h), thus natural daily and seasonal fluctuations of seawater parameters (e.g., temperature, pH, salinity, nutrients) were transferred to the benthocosms (units with ambient temperature were adjusted equally to the actual seawater temperature in the Kiel Fjord). Water temperature in the tanks was constantly monitored by internal sensors and automatically controlled via a heating/cooling system. The benthocosms were covered with slanted, translucent hoods resulting in independent gas-tight headspaces per unit. The *p*CO_2_ in the headspace was constantly monitored by internal gas sensors and manipulated by injections of pure CO_2_ to a defined concentration, causing seawater acidification by atmosphere-water gas exchange. The latter was enhanced by wave generators (seawater inflow) and aspirator pumps, installed to mimic water movements similar to Baltic coastal zones and to enlarge the mixing efficiency.

### Experimental Setup and Treatments

Within 1 year, four consecutive seasonal experiments were performed during April 2013 to March 2014 as described by Wahl et al. (2015a, 2019) for 10–11 weeks: spring (04/04, 05/02, 05/30, and 06/19/2013, 11 weeks), summer (07/04, 08/01, 08/29, and 09/17/2013, 11 weeks), autumn (10/10, 11/07, 12/05, and 12/17/2013, 10 weeks) and winter (01/16, 02/13, 03/13, and 04/01/2014, 11 weeks). For each experiment, stones bearing at least one *F. vesiculosus* individual were collected along the Kiel Fjord (at 54°27′14.951′′N 10° 11′ 51.886′′E) at a depth of ∼1 m (for a detailed description of the sampling site see Graiff et al., 2015). Grazers, attached to the *F. vesiculosus* individuals that were used for biofilm sampling, were carefully removed in seawater to keep the natural microbiota. Each benthocosm unit was populated with 20 (25 for spring experiment) stones carrying *F. vesiculosus* and several organisms commonly found in the natural habitat of Baltic *F. vesiculosus* (main mesograzers: the periwinkles *Littorina littorea*, *Idotea* spp. isopod crustaceans and *Gammarus* spp. amphipods, see Werner et al., 2016). The amount of grazers varied between seasons due to their natural abundance fluctuations in the Kiel Fjord (Werner et al., 2016), however, macroalgal and grazer biomass was equally distributed to the 12 units in each experiment. Stones with *F. vesiculosus* were placed at fixed positions onto the gratings at ∼40 cm water depth. *F. vesiculosus* individuals were incubated in the benthocosms under one of four different conditions: (1) increased temperature (+5°C temperature at ambient *p*CO_2_), (2) elevated *p*CO_2_ (1100 μatm *p*CO_2_ at ambient temperature), (3) increased temperature and elevated *p*CO_2_ (+5°C temperature at 1100 μatm *p*CO_2_), and (4) ambient (ambient temperature and ambient *p*CO_2_) with three units per treatment.

### Temperature and pH/*p*CO_2_

As described elsewhere (Graiff et al., 2015; Wahl et al., 2015a) seawater parameters were documented regularly: temperature, pH, salinity, and O_2_ were constantly logged by sensors, in addition daily hand-held pH measurements were performed, total alkalinity (TA) and nutrients (PO_4_, NO_3_) were measured twice per week, and dissolved inorganic carbon (DIC) monthly. The original raw data of constantly logged temperature and pH data per benthocosm unit are available at the PANGAEA^®^ data platform^1^.

Time courses of temperature and pH values for all treatments of all four benthocosm experiments are shown in Supplementary Figure S2 and in related publications (Graiff et al., 2015; Wahl et al., 2015a). Supplementary Figure S3 shows the salinity of the Kiel Fjord seawater that was constantly supplied to the benthocosms.

### Sampling of *F. vesiculosus* Biofilm and Surrounding Seawater

Seawater samples were taken via side ports at ∼20 cm water depth (while hoods were still closed to keep the adjusted atmosphere during the sampling event) into sterile 2 L bottles, transferred to the laboratory and kept at 4°C in the dark until vacuum-filtered within 1 day. Water was pre-filtered through 10 μm Isopore polycarbonate membrane filters (Millipore, Billerica, MA, United States) to remove particles larger than 10 μm, and subsequently planktonic cells from 1 L were collected by vacuum filtration through 0.22 μm Millipore Express PLUS polyethersulfone membrane filters (Millipore, Billerica, MA, United States) at max. −0.2 bar. Membrane filters were transferred to 2 mL reaction tubes and subsequently frozen in liquid nitrogen.

The hoods were opened shortly before the biofilm sampling. Prior to biofilm sampling, the *F. vesiculosus* thalli were rinsed with freshly prepared 0.22 μm filtered artificial seawater (17 g/L, Reef Crystals, Aquarium Systems, Sarrebourg, France) with a salinity of 15 practical salinity units (PSU), comparable to the Kiel Fjord seawater salt concentrations, to remove non-attached cells and particles. Importantly, during each experiment biofilm samples were repeatedly taken from the upper, younger parts of 2–3 thalli (∼15 cm^2^ by visual estimation) of the same *F. vesiculosus* individuals (one *F. vesiculosus* per unit at a fixed position where the action of the wave generator mixed the water). For each sampling event biofilm samples were taken from different thallus tips of the same individual. Sampled thallus areas were subsequently labeled with small cable straps to prevent resampling of the same region. The biofilm samples were taken with sterile cotton tips, transferred to 2 mL reaction tubes and subsequently frozen in liquid nitrogen. All shock-frozen samples were stored at −80°C until DNA extraction.

Samples were taken after 0, 4, 8, and 10, or 11 weeks of incubation per seasonal experiment (*n* = 3 per treatment), with two exceptions: (1) missing biofilm data due to high fouling of *F. vesiculosus* under warming during summer in week 11 and (2) missing water data due to canceled filtration because of the storm “Xaver” (5.-6.12.2013) during autumn in week 8.

### DNA Extraction and PCR Amplification of Bacterial 16S rDNA for Illumina MiSeq Amplicon Sequencing

Genomic DNA was isolated using the Isol-RNA Lysis Reagent (5 PRIME, Gaithersburg, MD, United States) as described by Mensch et al. (2016). The PCR amplification of the hypervariable region V1–V2 of the bacterial 16S rDNA, the subsequent purification and pooling of the amplicons, as well as the Illumina MiSeq sequencing were performed as described in Mensch et al. (2016). Sequences were submitted to the NCBI Sequence Read Archive under accession number SRP075254.

### Bioinformatic Processing

Raw data of two MiSeq runs were combined and a quality filter on raw reads was performed using the trimmer program Trimmomatic version 0.33 (Bolger et al., 2014). Briefly, reads were analyzed with a sliding window of 4 bp. Regions were trimmed if the average Phred score (Ewing and Green, 1998) within the window was below 30. Illumina adapters and primer sequences were removed. Trimmed reads were kept within the dataset if the forward and reverse read both survived the quality trimming and were longer than 36 bp. Sequence processing was continued using Mothur version 1.35.1 (Schloss et al., 2009; Kozich et al., 2013) according to Mensch et al. (2016) as follows: trimmed reads were concatenated to 31,838,465 contiguous sequences (contigs) using the command *make.contig*. Contigs with ambiguous bases or homopolymers longer than 8 bases as well as contigs longer than 552 bases were removed (*screen.seqs*). The remaining 20,636,961 contigs were screened for redundant sequences (*unique.seqs*) and clustered into 1,642,619 unique sequences. The sequences were consecutively aligned (*align.seqs*) to a modified version of the SILVA database release 102 (Pruesse et al., 2007) containing only the hypervariable regions V1 and V2. Sequences not aligning in the expected region were removed from the dataset (*screen.seqs*). The alignment was condensed by removing gap-only columns (*filter.seqs*). The final alignment contained 19,311,894 sequences (1,126,879 unique) of lengths between 244 and 401 bases. Command *pre.cluster* was used to include sequences with up to three positional differences compared to larger sequence clusters into the latter. Chimeric sequences were removed using the Uchime algorithm (Edgar et al., 2011) (*chimera.uchime*, followed by *remove.seqs*). This left 12,575,261 sequences (205,946 unique) in the dataset. Sequence classification was performed with *classify.seqs* using the Wang Method (Wang et al., 2007) on a modified Greengenes database (containing only the hypervariable regions V1 and V2) with a bootstrap threshold of 80%. Sequences belonging to the kingdom archaea, to chloroplasts or mitochondria were removed from the dataset (*remove.lineage*). OTUs were formed using the average neighbor clustering method with *cluster.split*. This step was parallelized by taking into account the taxonomic classification at the order level. A sample-by-OTU table containing 57,197 OTUs at the 97% level was generated (*make.shared*). OTUs were classified taxonomically using the modified Greengenes database mentioned above (*classify.otu*).

### Statistical Analysis of Bacterial 16S rDNA Amplicon Data

Statistical downstream analysis was conducted with custom scripts using R (v.3.3.1) as recently outlined by Mensch et al. (2016). OTUs of very low abundance only increase computation time without contributing useful information thus were removed from the dataset, resulting in a decrease in the number of OTUs from 57,197 to 3,095 retained in the filtered OTU table, while still representing 95% of the overall OTU counts. The median (IQR) overall OTU count per sample was 7605 (4739–12822). A redundancy analysis (RDA) was performed with Hellinger-transformed OTU counts as described by Mensch et al. (2016) to explore the extent of change in relative OTU abundance across samples explained by the experimental factors Temperature, *p*CO_2_, Week and habitat Type per Season (see metadata Supplementary Table S1). The latter (subset data “Season”) was performed to distinguish subset RDA models per season, firstly because different *F. vesiculosus* individuals were used in the four experiments and secondly due to some missing sequencing data excluded from the RDA [summer week 11 (decayed macroalgae) and autumn week 8 (storm)]. The factor *p*CO_2_ was removed from the RDA model at an early stage, because the *p*CO_2_ effect was not statistically significant (significance threshold *p* < 0.05). The temperature effects per season and type were visualized using the R package *ggplot2* v3.1.1 (Wickham, 2016).

Further analyses were conducted as recently described by Mensch et al. (2016). OTUs significant at an false discovery rate of 5% (*q* ≤ 0.05) were further subject to indicator analysis with function *multipatt* of the R package *indicspecies* v1.7.5 (Cáceres and Legendre, 2009) with 10^5^ permutations. Indicator OTUs (iOTUs) – in analogy to indicator species *sensu* Cáceres and Legendre (2009) – are OTUs that prevail in a certain sample group (here: a level of Temperature within a certain sample Type and level of Week) while being found only irregularly and at low abundance in other sample groups.

Venn diagrams of OTU shared between sample groups were created with R package *VennDiagram* v1.6.20 (Chen, 2018). For alpha diversity analysis, effective OTU richness [Shannon numbers equivalent, ^1^D (Jost, 2006, 2007)] was calculated from the filtered OTU table. Multi-panel alpha diversity and iOTU plots were drawn with R package *lattice* v0.20-33 (Sarkar, 2008). An OTU association network (incorporating all sequenced samples due to small datasets per season) was inferred with the R package *SpiecEasi* v0.1 (Kurtz et al., 2015). OTUs were required to be present in ≥60% of all samples to reduce the number of zeros in the data for a robust calculation, resulting in a set of 42 OTUs. Calculations were performed with the Meinshausen and Bühlmann neighborhood selection framework (MB method, Meinshausen and Bühlmann, 2006). Correlations between associated OTUs were determined from the centered log-ratio-transformed counts. The *igraph* package v1.0.1 (Csárdi and Nepusz, 2006) was employed for visualizing strong OTU associations (absolute correlation ≥ 0.6).

## Results

During April 2013 to March 2014 four consecutive seasonal experiments were performed as described in detail in Wahl et al. (2015a, 2019) and the “Materials and Methods” section, to monitor changes in the bacterial communities associated with *F. vesiculosus* in response to acidification and warming. During the experiments the monthly mean ambient seawater temperature increased from 8.2 ± 1.4 to 16.1 ± 1.3°C in the spring experiment (Supplementary Figure S2; see also Graiff et al., 2015; Wahl et al., 2015a). During summer, maximum temperatures were recorded in late July–August (19.5 ± 1.2, max. 24.8°C), followed by a decrease to 17.4 ± 1.0°C in September. Then, ambient seawater temperature decreased from 13.1 ± 0.5 to 6.9 ± 0.5°C in autumn and slightly increased again from 4.2 ± 1.4 to 6.7 ± 1.1°C in winter. In each season, seawater temperature was approximately +5°C higher in the warming treatments. Importantly, during the summer experiment in late July to August daily temperatures of ∼29°C were reached in the warming treatments. In general, ambient temperature showed a seasonal variation typical for the region with a minimum of about 1°C in late January/early February and a maximum of about 24°C in late July/early August. In addition, daily fluctuations in the order of 1–3°C were observed, reaching extremes of 5°C on sunny summer days (Wahl et al., 2015a). During the experiments the monthly mean ambient seawater pH (NBS scale, Supplementary Figure S2; Graiff et al., 2015; Wahl et al., 2015a) ranged from 8.73 ± 0.11 to 8.26 ± 0.39 in spring, from 8.20 ± 0.19 to 7.91 ± 0.12 in summer, from 7.97 ± 0.09 to 7.87 ± 0.05 in autumn and from 7.96 ± 0.07 to 8.08 ± 0.14 in winter, with the overall tendency of constantly declining pH values over the year. Highest pH values were measured in April at the beginning of the spring experiment and lowest pH values in December at the end of the autumn experiment, then again rising during the winter experiment. Atmospheric CO_2_ enrichment of elevated *p*CO_2_ treatments (1050–1100 μatm) resulted in a pH reduction of the seawater by 0.18 ± 0.08 compared to ambient *p*CO_2_ conditions (380–450 μatm, free air-circulation under hood; Wahl et al., 2015a). All benthocosms were constantly supplied with seawater from the Kiel Fjord. The salinity changed during the year from 12.4 ± 0.3 in April to highest 20.7 ± 1.5 PSU in December 2013 (Supplementary Figure S3).

### (I) Bacterial Community Composition of *F. vesiculosus* Biofilm and Seawater

#### Temporal Development of the Bacterial Communities

**FIGURE 1:**
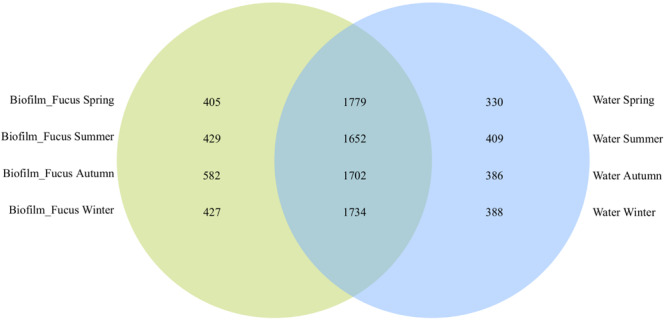
OTU overlaps among biofilm and seawater bacterial communities per season. Venn diagram illustrating the counts of shared OTUs between *Fucus vesiculosus* biofilm (“Biofilm_Fucus”, green) and seawater (“Water”, blue) bacterial communities during spring, summer, autumn, and winter.

**FIGURE 2:**
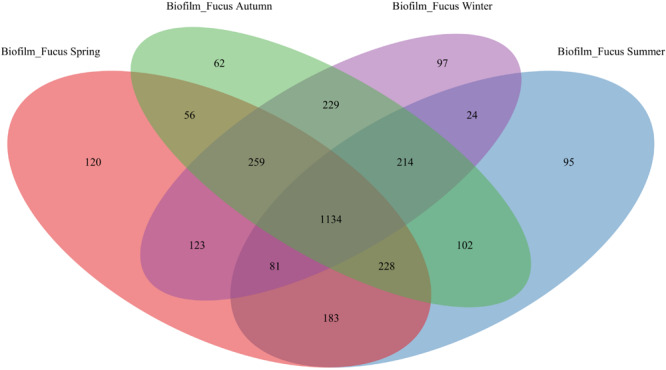
OTU overlap of biofilm bacterial communities among seasons. Venn diagram illustrating the counts of OTUs shared by *Fucus vesiculosus* biofilm (“Biofilm_Fucus”) bacterial communities among spring, summer, autumn, and winter.

**FIGURE 3:**
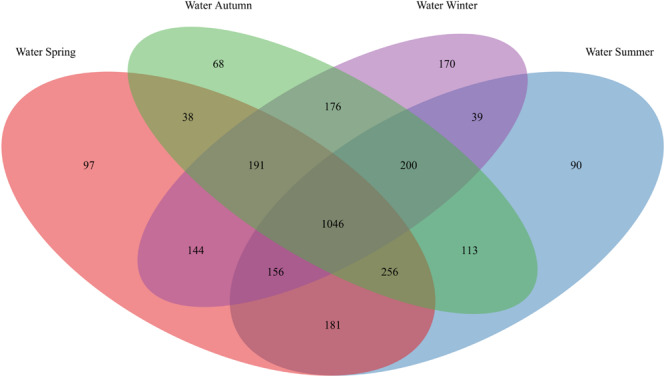
OTU overlap of seawater bacterial communities among seasons. Venn diagram illustrating the counts of OTUs shared by seawater (“Water”) bacterial communities among spring, summer, autumn, and winter.

The majority of OTUs were shared among biofilm and seawater bacterial communities during all seasons (1717 ± 46 OTUs), however, more OTUs were specific to the surface of *F. vesiculosus* than were specific for the surrounding seawater (Figure 1). On *F. vesiculosus* 405, 429, 582, and 427 OTUs were detected during the spring, summer, autumn, and winter experiments, respectively. The Figures 2, 3 provides the numbers of OTUs shared among all seasons within the biofilm and seawater samples, respectively.

At the OTU level, the bacterial communities varied in their composition depending on habitat type (biofilm or seawater) and on season (spring, summer, autumn, or winter). Within each season, the bacterial communities on *F. vesiculosus* and in the surrounding seawater developed over weeks 0, 4, 8, and 10/11 remarkably (*F* ≥ 2) differing in their community composition as revealed by pairwise *F*-value clustering (Supplementary Figures S4, S5), respectively. An exception was observed in winter, when differences in biofilm community patterns between weeks were hardly (week 0 vs. 4) or not significant (week 8 vs. 11) (Supplementary Figure S4D).

#### Phylogenetic Composition of the Bacterial Communities

Across all seasons and treatments, the biofilm attached to the *F. vesiculosus* surface was generally dominated by *Proteobacteria* (mainly *Alpha-* and *Gamma*-, fewer *Delta-* and *Betaproteobacteria*), *Bacteroidetes* (*Flavobacteriia*, *Saprospirae*, and *Cytophagia*), *Actinobacteria* (*Actinobacteria* and *Acidimicrobiia*), and *Cyanobacteria* (*Oscillatoriophycideae*) (Supplementary Figure S6). However, the epibacterial community composition was highly variable between the *F. vesiculosus* replicates, and the relative abundances of bacterial classes varied between weeks and seasons (*n* = 3 per treatment; 180 biofilm samples in total).

Under ambient conditions, the majority of the epibacterial community on *F. vesiculosus* consisted of *Alphaproteobacteria*, *Gammaproteobacteria*, *Flavobacteriia*, and *Saprospirae*, depending on week and season (spring 61.0 ± 13.5%, summer 49.0 ± 9.1%, autumn 51.1 ± 9.6%, and winter 65.4 ± 6.4%, mean ± SD% relative abundance). During spring, the *Alpha-* and *Gammaproteobacteria* together accounted for 43.7 ± 11.8%, whereas their proportion was lower in the other seasons (36.0 ± 6.7% in summer to winter). In contrast to spring to autumn (16.7 ± 10.6%) *Flavobacteriia* and *Saprospirae* together tended to be more abundant in the biofilm during winter (25.3 ± 6.7%). During autumn to winter 10.4 ± 4.8% *Deltaproteobacteria* were present on *F. vesiculosus*, about twice as much than found in spring to summer (5.6 ± 2.9%). *Betaproteobacteria* showed irregular pattern in single biofilm samples during spring to summer with 8.6 ± 12.5% and were nearly absent in autumn to winter (1.5 ± 1.8%). *Acidimicrobiia* were nearly absent in spring (1.4 ± 0.9%), but low abundant through the rest of the year (3.8 ± 2.5%), and *Cytophagia* were hardly found in winter (0.9 ± 0.5%), however, showed small abundance in the other seasons (3.2 ± 2.3%). Compared to summer and winter (0.5 ± 0.9%) the class *Oscillatoriophycideae* showed its highest abundances in spring and autumn (3.0 ± 2.4% and 2.0 ± 4.2%, respectively), whereas the *Actinobacteria* were least abundant through all seasons (0.7 ± 1.1%). At the end of the summer experiment (week 11), only few biofilm samples were taken because water temperatures of ∼24°C and ∼29°C were reached for ambient and increased temperature treatments, respectively, both resulting in the death of the host algae (see “Materials and Methods” section); in particular under the warming treatment *F. vesiculosus* was completely decayed.

Under ambient conditions, the majority of the free-living bacteria in the surrounding seawater belonged to *Alpha*-, *Gammaproteobacteria*, and *Flavobacteriia* (Supplementary Figure S7). Although varying in proportion, together these three classes accounted for 75.3 ± 11.8% of the total bacterial community composition in spring, summer and winter, contrarily to 55.9 ± 12.7% in autumn. Among them, the *Flavobacteriia* reached highest prevalence during summer (24.1 ± 14.0%). Furthermore, the proportion of *Actinobacteria* was markedly higher in autumn (19.2 ± 14.8%) than in all other seasons (4.6 ± 3.1%). *Betaproteobacteria* were constantly present (5.8 ± 3.8%) throughout all seasons. *Saprospirae* were nearly absent in summer and reached highest proportion during spring (1.2 ± 1.6% and 8.8 ± 15.0%, respectively), whereas *Deltaproteobacteria* were constantly low abundant though all seasons (1.3 ± 1.2%) and *Acidimicrobiia* showed highest percentage during autumn (4.7 ± 2.8%). In week 8 of the autumn experiment, seawater samples were lost due to a severe storm (see “Materials and Methods” section; *n* = 3 per treatment; 180 water samples in total). In general, contrarily to the high variation in community composition in *F. vesiculosus* biofilms, the variation in community composition between replicates of the surrounding seawater was smaller.

#### Alpha Diversity

**FIGURE 4:**
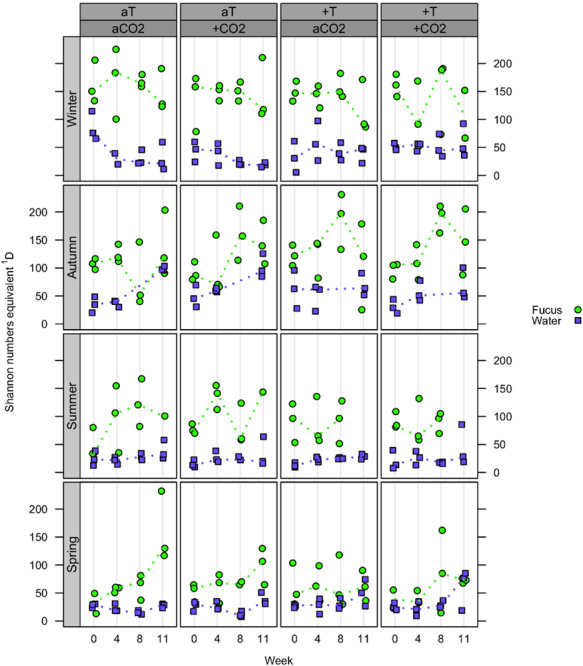
Effective OTU richness (alpha diversity). Distribution of Shannon numbers equivalents ^1^D at OTU level concerning *Fucus vesiculosus* biofilm (green circles) and water samples (blue squares) after 0, 4, 8, and 10/11 weeks of treatment during spring, summer, autumn, and winter, respectively, in the Kiel Benthocosms at four different conditions (see section “Materials and Methods”; T, temperature; CO_2_, *p*CO_2_; a, ambient; +, increased). Dots represent ^1^D of each single sample and dotted lines connect the medians (*n* = 3) between weeks.

At the OTU level, the effective OTU richness ^1^D (or Shannon numbers equivalents, expressing the alpha diversity; Jost, 2006, 2007) of *F. vesiculosus* biofilm tended to be higher in autumn and winter (up to median values of ∼200) compared to spring and summer (up to median values of ∼150), however, with generally high variability between biofilm replicates (Figure 4). Further, in spring and autumn, biofilm ^1^D tended to increase over time. In the surrounding water, ^1^D was not affected by season and showed small variation at constantly low median values (∼25 in spring and summer, and ∼50 in autumn and winter). In general, ^1^D was much higher in biofilm than in seawater samples. No clear differences in ^1^D were observed in the single and combined *p*CO_2_ or warming treatments for both habitat types in all seasons.

### (II) OTU Association Network

**FIGURE 5:**
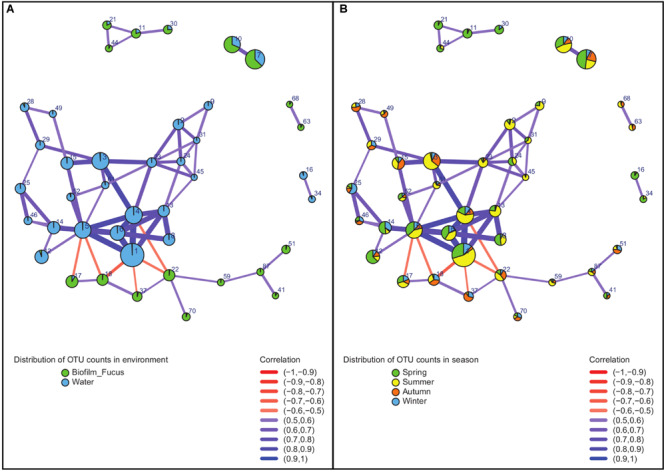
Association network of strongly correlated OTUs. Analysis was performed on sequence data of entire dataset. Vertices represent OTUs and are labeled by OTU number (for details see Supplementary Table S2) and colored according to **(A)** distribution in environment (habitat) or **(B)** distribution in season, respectively. Width and color of the edges connecting the vertices vary according to strength and sign (color gradient red to blue describes negative to positive) of the correlation between associated OTUs. Diameters of the vertices vary according to the relative abundance of each OTU.

Bacterial correlations (within the entire dataset of 366 sequenced samples including treatment samples) described by an OTU association network of OTUs that were consistently present across the majority (≥60%, see “Materials and Methods” section) of samples included 42 OTUs with moderate to strong correlations (Figure 5 and Supplementary Table S2). Their strength and type of correlation, as well as environmental (habitat related) and seasonal distribution within the OTU network are shown in Figure 5. The network-forming OTUs belonged to 11 bacterial classes with the majority belonging to *Alphaproteobacteria*, *Flavobacteriia*, *Beta-* and *Gammaproteobacteria*, complemented by OTUs of *Saprospirae*, *Acidimicrobiia*, *Actinobacteria*, *BME43* cluster, *Deltaproteobacteria*, *Mollicutes*, and *Oscillatoriophycideae* (Supplementary Table S2). Apart from few exceptions, all network OTUs were either found in biofilm or water samples (Figure 5A): positive correlations appeared only between OTUs dominating the same habitat, and negative correlations were rare and occurred solely between OTUs of different habitats. The proportional distribution of network-forming OTU counts varied with season (Figure 5B): Largest proportions were found in summer and spring, less in autumn, and smallest in winter. The OTU association network analysis (Figure 5 and Supplementary Table S2) revealed some notable findings described in the following section.

One of the rare network OTUs present in both habitats, biofilm and seawater, was OTU#30 (*Glaciecola punicea*) found in spring, and to a small extent in winter. This OTU occurred to ∼25% in the surrounding seawater and to ∼75% associated with *F. vesiculosus*. OTUs found to be primary attached to *F. vesiculosus* were the positively correlated OTU#87 (*Rubidimonas crustatorum*) and OTU#41 [unclassified (uncl.) *Maribacter*]. Both were present in all seasons, but dominant in summer and spring. Further, OTU#17 (uncl. *Erythrobacter*) and OTU#18 (uncl. *Polyangiaceae*) were also positively correlated in biofilm samples. They occurred in all seasons, but OTU#17 was dominant in spring to summer, and OTU#18 was present mainly in summer to winter. Additionally correlated OTUs in the *F. vesiculosus* biofilm were OTU#37 (uncl. *Thiohalorhabdales*), OTU#70 (acidimicrobial *C111*), OTU#59 (uncl. *Flavobacteriaceae*), and OTU#51 (uncl. *Hyphomonadaceae*).

Negative correlations between OTUs of different habitats were found, for instance, between both, OTU#17 and OTU#18 (positive correlated uncl. *Erythrobacter* and uncl. *Polyangiaceae*) predominantly on *F. vesiculosus*, and OTU#5 [uncl. “*Candidatus* (*Ca.*) Portiera”] in seawater, as well as between OTU#18 (uncl. *Polyangiaceae*) on *F. vesiculosus* and OTU#1 (*Pelagibacter ubique*) in seawater. Moreover, OTU#1 was strongly positive correlated with three OTUs (#4, #6, #13) in water of the same family *Pelagibacteraceae* underlining the essential role of this abundant family for microbial seawater communities. Additionally, OTU#1 (*P. ubique*) and OTU#5 (uncl. “*Ca.* Portiera”) were positively correlated in seawater, both present in all seasons, but mainly in summer and spring. Another example for positively correlated OTUs in water were OTU#31 (“*Ca.* Aquiluna rubra”) and OTU#24 (uncl. *Acholeplasma*), both present in spring and summer. Further OTUs predominantly associated with seawater samples were OTU#3 (uncl. *Octadecabacter*) detected in all seasons, but mainly summer and autumn, as well as OTU#34 (uncl. *RS62*, *Comamonadaceae*) mainly found in spring and less in summer.

### (III) Temperature and *p*CO_2_ Effects on Biofilm and Seawater Bacterial Communities per Season

**FIGURE 6:**
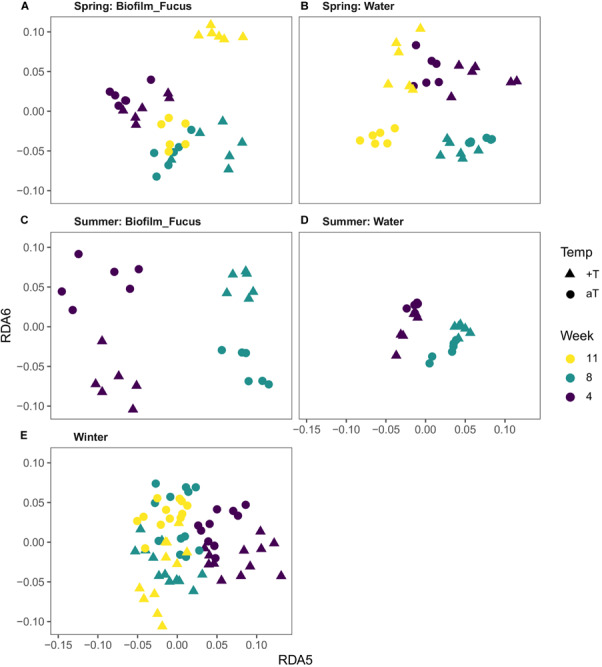
Visualization of the temperature effect on bacterial communities. Distance plots were derived from respective RDA models (see “Materials and Methods” section). Since the temperature effect is rather subtle, higher dimensions (constrained axes “RDA 5” and “RDA 6”) were chosen to achieve optimal visual differentiation between temperature treatment groups (Temp; aT, ambient temperature; +T, increased temperature). In spring **(A,B)** and summer **(C,D)**, the difference in community composition depends on the origin of the bacterial communities (“Biofilm_Fucus” or “Water”) as well as on the duration of the treatment (Week; note the different directions in which the groups are separated). In winter **(E)**, samples are uniformly separated according to temperature treatment, regardless of duration and origin.

Redundancy analysis (RDA) was performed as described in the “Materials and Methods” section, showing that a restructuring of bacterial community structures to elevated *p*CO_2_ levels was not statistically significant in any season. Further, no additional interaction of elevated *p*CO_2_ with increased temperature was observed. However, the impact of increased temperature on the bacterial communities varied between the seasons, but could not be measured in autumn (Figure 6 and Supplementary Table S3).

In spring, a week-wise evaluation revealed that biofilm and water communities significantly developed over time while both habitat types were differently affected by increased temperature (Type:Temp:Week interaction, Supplementary Table S3). In weeks 4, 8, and 11, increased temperature significantly restructured the biofilm [*F*_(1,10)_ = 1.75, *p* = 0.026; *F*_(1,10)_ = 2.18, *p* = 0.005; and *F*_(1,10)_ = 1.89, *p* = 0.007] and seawater communities [*F*_(1,10)_ = 2.84, *p* = 0.007; *F*_(1,10)_ = 3.44, *p* = 0.006; and *F*_(1,10)_ = 3.04, *p* = 0.007], see Figures 6A,B. Although the temperature effect on biofilm was statistically weak (indicated by *F* ≤ 2) in weeks 4 and 11, it was highly significant. The temperature effect in weeks 4, 8, and 11 on biofilm and water samples explained 6, 10, and 7% and 14, 18, and 16% of total variance in spring, respectively.

In summer, the bacterial communities of both habitat types significantly developed over time while they were differently affected by the warming treatment (Type:Temp:Week interaction, Supplementary Table S3), shown by a week-wise evaluation. In weeks 4 and 8, increased temperature significantly reshaped the biofilm [*F*_(1,10)_ = 2.79, *p* = 0.003; and *F*_(1,10)_ = 4.15, *p* = 0.001] and water communities [*F*_(1,10)_ = 2.00, *p* = 0.005; and *F*_(1,10)_ = 2.97, *p* = 0.002], see Figures 6C,D. Remarkably, the temperature effect on biofilm samples was stronger in summer than in spring, with strongest effect size (*F* value) in week 8 [Biofilm.Temp.Week08, *F*_(1,10)_ = 4.15, *p* = 0.001]. [No data is available for week 11 due to collapsed system during hot summer months (see “Materials and Methods” section).] The temperature effect in weeks 4 and 8 on biofilm and water samples explained roughly 14 and 22% and 8 and 15% of total variance in summer, respectively. A weak, but significant temperature effect on biofilm at the beginning of the summer experiment was measured [Biofilm.Temp.Week00, *F*_(1,10)_ = 1.68, *p* = 0.031], however, the iOTU analysis revealed negligible results. This effect might be most likely traced back to a technical artifact of the benthocosm facility, namely to approximately 0.5°C temperature difference between ambient and warm tanks at the beginning of each experiment shortly before treatment start.

No temperature effect could be measured in autumn. However, a week-wise evaluation demonstrated that both types significantly differed in their composition since experimental start, and further developed differently over time (*p* < 0.001 for each week-wise comparison of weeks 0, 4, and 10, respectively) with the most remarkable differences between start and end of the experiment [Biofilm Week00 – Week10, *F*_(1,22)_ = 6.53, *p* < 0.001; Water Week00 – Week10, *F*_(1,22)_ = 7.38, *p* < 0.001; Type:Week interaction, Supplementary Table S3]. Roughly, the week effect among biofilm and water samples explained 8–19% and 13–22% of total variance in autumn, respectively.

Although the temperature effect in winter explained only 1% of total variance, the RDA revealed a highly significant superior temperature effect that affected both habitat types similarly in each week [Temp, *F*_(1,85)_ = 2.17, *p* = 0.001; Temp + Type:Week interaction (=significant Temperature term and significant Type:Week interaction); Supplementary Table S3], see Figure 6E. An interaction of Temperature with Week was not statistically significant, but was assumed since the pairwise *F*-value clustering showed that biofilm and water communities markedly (*F* > 2) differed between weeks thus developed over time [*p* = 0.001 per week-wise comparison; Biofilm.Week08 ∪ 11 were exceptionally unified (∪) due to high similarity, Supplementary Table S3, see also Supplementary Figures S4, S5]. Similar to autumn, during winter the most remarkable differences in community composition were observed between start and unified weeks 8/11 of the experiment for both types, respectively [Biofilm Week00 – Week08 ∪ 11, *F*_(1,32)_ = 6.53; Water Week00 – Week08/11, *F*_(1,21)_ = 7.48/7.24]. On the basis of a week-wise comparison, the week effect among biofilm and water samples explained roughly 6–14% and 7–23% of total variance in winter, respectively.

### (IV) Temperature Related iOTUs in Spring, Summer, and Winter

The temperature effects in spring, summer and winter could be traced back to single OTUs that significantly differ in their relative abundance between ambient (aT) and increased (+T) temperature levels (false discovery rate of *q* ≤ 0.05). According to that, an iOTU for aT is more abundant at ambient temperature conditions and an iOTU for +T shows higher relative abundance at increased temperature. Table 1 summarizes the counts of iOTUs for temperature detected per season, week and type. During summer, the number of iOTUs for +T on *F. vesiculosus* remarkable increased from 12 to 38 in week 4 to 8. The temperature effect on the bacterial communities was stronger in summer than in spring, supported by higher effect sizes from statistical testing (see section “Results” part III for related *F*-values) and the observation that on *F. vesiculosus* roughly twice as much classes were found to harbor iOTUs that were affected by temperature in summer (17 classes) compared to spring (9 classes). To gain more detailed insights into the complex restructuring of bacterial communities in response to warming in spring, summer and winter, Supplementary Tables S4, S5 comprises all iOTUs for aT and +T, respectively, with taxonomic information at least at genus level. The mean differences in relative abundance (Δ‰ = ‰ +T - ‰ aT) underline the negative (–) or positive (+) impacts of the applied warming on a certain iOTU.

**TABLE 1:** Counts of indicator OTUs for temperature. Counts of iOTUs for ambient (aT) and increased temperature (+T; treatment details see “Materials and Methods” section) detected in *Fucus vesiculosus* biofilm and seawater bacterial communities during spring (weeks 4, 8, and 11), summer (weeks 4 and 8), and winter, respectively.

		**Spring**			**Summer**		**Winter**
		**Week 4**	**Week 8**	**Week 11**	**Week 4**	**Week 8**	
aT	Biofilm	14	5	22	26	29	34
	Water	0	8	4	16	10	
+T	Biofilm	1	3	2	12	38	44
	Water	1	10	0	12	7	

#### Selected Indicators Varying Between Both Temperature Levels (Compare Supplementary Tables S4, S5)

On lower taxonomic resolution, many taxa included single iOTUs for both temperature levels (mainly with different OTU numbers) varying with season, week and/or type, because the majority of all iOTUs could not exactly be specified by the performed amplicon sequencing. On *F. vesiculosus*, the genus *Erythrobacter* was indicator for aT in spring and summer, and further for +T in winter. Surface attached to *F. vesiculosus* in summer were iOTUs of the genus *Roseivirga* as indicators for +T and aT. Indicators for both temperature levels in summer on *F. vesiculosus* belonged to *Crocinitomix*, also detected as iOTU for +T in winter. *Maribacter* was indicator for +T in summer on *F. vesiculosus*, but indicator for aT in winter. In seawater, the genus *Sediminicola* was indicator for aT in spring, but for +T in summer. The genus *Octadecabacter* was indicator for aT in summer in seawater, however, the species *O. antarcticus* was iOTU for +T in winter. In winter, the genera *Polaribacter* and *Fluviicola* were indicators for both +T and aT, respectively. Three species of the genus *Glaciecola* were identified to be indicators for temperature in different seasons and weeks, associated with both habitat types, while the majority was indicator for aT. *G. punicea* (iOTU for aT on *F. vesiculosus* during spring in week 8 with Δ-50.19‰ and in week 11 with Δ-16.52‰), *G. mesophila* (iOTU for aT in water during summer in week 8 with Δ-2.37‰; but iOTU for +T in winter with Δ+10.89‰) and *G. psychrophila* (iOTU for aT in winter with Δ-0.69‰). In addition, further different iOTUs belonging to the genus *Glaciecola* could not be specified, but were indicators for aT in summer for *F. vesiculosus* (Δ-15.16‰) and in winter for both types, as well as iOTUs for +T and aT in seawater (Δ-20.18‰) during summer

#### Selected iOTUs for Ambient Temperature (aT) Level (see Supplementary Table S4)

Among the iOTUs for aT on *F. vesiculosus* in spring were *Psychroserpens mesophilus*, as well as the genera *Luteolibacter* and *Rickettsia*. In summer, *Hirschia baltica*, *Anabaena cylindrica* (in week 4 with Δ-9.88‰) and *R. crustatorum* (in week 8 with Δ-9.66‰) were identified as iOTUs for aT associated with *F. vesiculosus*. The genus *Flavobacterium* was detected as iOTUs for aT in spring for both types and during summer in seawater. *Lewinella* comprised iOTUs for aT in spring on *F. vesiculosus* and in winter. In seawater *P. ubique* (in week 8 with Δ-190.14‰ based on 480.14‰ at aT vs. 290.00‰ at +T) and “*Ca.* Aquiluna rubra” were iOTUs for aT in spring. In summer, iOTUs for aT in water belonged to the genera *Coraliomargarita*, “*Ca.* Portiera,” gammaproteobacterial *HTCC2207*, *Microbacterium*, *Pseudomonas* and *Yonghaparkia*. The genus *Persicirhabdus* was an indicator for aT in water in summer, and was also detected at aT in winter. In winter, the genus *Roseibacillus* was also identified as indicator for aT. The higher abundance of these iOTUs at ambient compared to increased temperature in turn means, that they suffered from warming.

#### Selected iOTUs for Increased Temperature (+T) Level (see Supplementary Table S5)

Although no specified iOTU for +T on *F. vesiculosus* could be identified in spring, an iOTU for +T attached to *F. vesiculosus* in summer was *Winogradskyella poriferorum* (Δ+3.21‰), in addition to iOTUs of the genera *Rivularia* and *Phormidium*. *Plesiocystis* was indicator for +T on *F. vesiculosus* during summer, and for both types in winter. In spring, iOTUs for +T in seawater belonged to the genera *Mycobacterium* and *Owenweeksia*, and in summer to *Thalassospira*, *Acholeplasma* and gammaproteobacterial *BD2-13*. In addition, the genus *Arcobacter* was indicator for +T in spring in water, and in summer iOTU associated with both *F. vesiculosus* and water. In winter, several iOTUs for +T belonged to the genera *Devosia*, *Leadbetterella*, *Formosa*, “*Ca.* Endobugula,” *Umboniibacter* and *Reichenbachiella*. In contrast to iOTUs for aT, iOTUs for +T benefited from warming.

## Discussion

### (I) Seasonal Variations in Epibacterial Communities of *F. vesiculosus*

The most abundant bacterial phyla attached to Baltic *F. vesiculosus* under ambient conditions were *Proteobacteria*, *Bacteroidetes* and *Actinobacteria*. In a comparable experimental design on Wadden Sea *F. mytili*, we observed a similar epibacterial community composition during spring that developed over time (Mensch et al., 2016). This is in agreement with previous field observations that the composition of epibacterial communities depends more on host identity than on habitat (North vs. Baltic Sea; Lachnit et al., 2009a). Although *F. mytili* and *F. vesiculosus* differ in many aspects regarding e.g., morphology (Albrecht, 1998) and the habitat conditions they live in (e.g., salinity, tidal ranges, nutrient concentrations), they are closely related macroalgal species. Thus, their surfaces were covered predominantly by the same bacterial classes during the spring experiments and their epibacterial communities only differ at finer phylogenetic resolution. Importantly, the same sampling method, DNA isolation and Illumina MiSeq sequencing technologies were used in our study on *F. mytili* (Mensch et al., 2016) supporting a reliable comparison of our studies. The epibacterial communities of Baltic *F. vesiculosus* showed temporal variability, as previously reported in a study using 16S rDNA cloning and Sanger sequencing (Lachnit et al., 2011), showing that biofilms naturally differ in their composition between seasons driven by changes in environmental conditions. Epibacterial communities of Baltic *F. vesiculosus* alter along temperature and salinity gradients (Stratil et al., 2013, 2014). During our experiments, salinity was monitored, and it changed during the course of the year (Supplementary Figure S3). However, salinity is not a defined experimental factor. While it is likely to have an additional influence on the bacterial communities, this effect was the same for all communities, since all benthocosms/treatments were constantly supplied with the same Fjord seawater. The salinity of the benthocosms and the Fjord were equivalent, shown by Wahl et al. (2015a).

Our findings on the epibacterial community composition of Baltic *F. vesiculosus* are in agreement with a previous study based on 16S rDNA amplicon pyrosequencing targeting the same hypervariable region V1-V2 (Stratil et al., 2013). Comparable to our previous study on the *F. mytili* holobiont (Mensch et al., 2016), at any given time point the biofilm replicates generally showed more variability between replicates than water samples, hinting at the influence of individual host physiology (or underlying genetic traits) and a better hydrodynamic homogenization of plankton within tanks and with the fjord water. In contrast, the biofilm community composition appeared more stable over time than seawater communities suggesting that *F. vesiculosus* controls to some degree its own microbiota via attracting and defending substances (Lachnit et al., 2009b; Wahl et al., 2010, 2012; Saha et al., 2011, 2012, 2017; Saha and Wahl, 2013) and that fluctuations in seawater conditions might affect free-living bacteria more directly (in contrast to matrix-embedded biofilm bacteria) resulting in a faster temporal response.

The alpha diversity (effective OTU richness) of the *F. vesiculosus* biofilm varied with season in response of epibacteria and/or host to shifts in seawater conditions in the course of the year. Similar to our previous study on Wadden Sea *F. mytili* (Mensch et al., 2016), the Baltic *F. vesiculosus* biofilm alpha diversity was about 4 (autumn/winter) to 6 times (spring/summer) higher than in the surrounding seawater, presumably because the macroalgal surface represents an attractive nutrient-rich habitat for settling and dense microbial life resulting in a higher species richness (Egan et al., 2013; Dang and Lovell, 2016).

### (II) Correlations Among Associated OTUs Mainly Governed by the Habitat

Positive correlations within OTU association networks suggest mutualistic interactions, while negative correlations often reflect competition (Steele et al., 2011; Faust and Raes, 2012; Fuhrman et al., 2015). However, negative correlations were exclusively found between OTUs dominating in either biofilm or seawater samples, verifying the habitat as most important factor in shaping the bacterial community compositions, as previously observed for *F. mytili* biofilm and North Sea water (Mensch et al., 2016). Strongest positive correlations were found between OTUs of the *Alphaproteobacteria*, the most abundant bacterial class on *F. vesiculosus* in this and other datasets (Stratil et al., 2013; Mensch et al., 2016; Parrot et al., 2019).

### (III) Bacterial Communities Were Affected by Temperature With Seasonal Variability, but *p*CO_2_ Had No Impact

Although a weak growth stimulating *p*CO_2_ effect was observed on Baltic *F. vesiculosus* hosts (Graiff et al., 2015), no direct *p*CO_2_ effects on biofilm and seawater bacterial communities in the single treatment, nor any interaction effect of *p*CO_2_ and temperature in the combined treatment was apparent in any seasons. The increase of *p*CO_2_ to 1100 μatm in the headspace resulted in a mean decrease of ∼0.2 pH units in the water column (Wahl et al., 2015a), representing only a small change in acidity for bacterial life under environmental conditions since most bacteria show a growth pH range of 2–3 units (e.g., neutrophiles 5.5–8.5 pH) (Madigan et al., 2009). In a similar spring experiment, we observed only a minor role of elevated *p*CO_2_ on bacterial communities on Wadden Sea *F. mytili* and in the surrounding North Sea water (Mensch et al., 2016). The alpha diversity of Baltic *F. vesiculosus* biofilm and seawater samples appeared not to be affected by the applied single and combined *p*CO_2_ and warming treatments across all seasons, consistent with previous results reported by Mensch et al. (2016).

Another interesting aspect is, that the microenvironment (including various types of microorganisms) of an algal surface is delimited by a diffusive boundary layer that is characterized by a fluctuating pH gradient. Extreme diel fluctuations about 1.5 pH units (i.e., 20 times stronger than the implemented acidification treatment) were measured within the epibacterial layer of Baltic *F. vesiculosus* (Wahl et al., 2015b). In addition, *F. vesiculosus* and its associated bacteria are already adapted to high fluctuations in pH/*p*CO_2_ conditions along the coasts of the western Baltic Sea including the Kiel Fjord (Melzner et al., 2013; Saderne et al., 2013).

The benthocosm experiments showed, that the macroalgal host *F. vesiculosus* suffered from warming in summer but was unaffected in the other seasons (also compare related *F. vesiculosus* tissue data of Graiff et al., 2015), whereas its biotic stressors (grazers and epiphytes) generally benefited from warming, except in summer when the grazers suffered but the epiphytes benefited (Graiff et al., 2015; Werner et al., 2016; Wahl et al., 2019). The bacterial communities on *F. vesiculosus* and in the surrounding seawater were affected by warming in all seasons but autumn. Since the biofilm is influenced by both its environment and its living host, the warming impacts on the epibacterial communities of *F. vesiculosus* might be a combination of direct effects on the biofilm and indirect effects via the macroalgal host. It has been shown that microfouling pressure by prokaryotes varied over the year, and chemical fouling control via surface metabolites of Baltic *F. vesiculosus* is fluctuating seasonally *in situ* with different abiotic factors (e.g., temperature) influencing the fouling control (Wahl et al., 2010; Saha and Wahl, 2013; Saha et al., 2014; Rickert et al., 2016a, b). In field studies, microfouling pressure was highest during summer (August) coinciding with a strengthened microfouling control observed from *F. vesiculosus* surface extracts (Rickert et al., 2016a, b). During summer, we noticed a threefold increase of iOTUs for +T on *F. vesiculosus*. Elevated temperatures during summer might disrupt the microfouling control in thermally stressed *F. vesiculosus* (Rickert et al., 2016a) resulting in an increase of bacteria related to intensified fouling and potential pathogenic species on the macroalgal surface.

### (IV) Several Bacteria of Potential Ecological Importance Suffered or Benefited From Warming

Among the iOTUs for ambient temperature were bacteria that were typically associated with a broad variety of marine hosts. Comparable high (≥Δ-10‰) differences in mean relative abundance between both temperature levels were detected among several iOTUs for ambient temperature, suggesting that these bacteria strongly suffer from warming, since they are dominant at ambient but (nearly) absent at the warming treatment. Among the iOTUs for ambient temperature on *F. vesiculosus* were the genus *Luteolibacter* known to include isolates from red algae (Yoon et al., 2008); the genus *Rickettsia* containing intracellular symbionts of eukaryotes reported for a wide range of eukaryotic hosts (Weinert et al., 2009); *Psychroserpens mesophilus* once isolated from a marine biofilm (Kwon et al., 2006); the budding bacterium *Hirschia baltica* originally isolated from brackish surface water of the Kiel Fjord (Schlesner et al., 1990) and *R. crustatorum* once isolated from a marine crustacean (Yoon et al., 2012). Additionally, *A. cylindrica* was markedly more abundant at ambient temperature on *F. vesiculosus*, in particular during summer when up to 29 and 34°C were reached in the ambient and increased temperature treatments, respectively. This in turn might confirm the temperature sensitivity of this nitrogen-fixing heterocystous cyanobacterium since under laboratory conditions increased temperatures of 37–40°C inhibited N_2_ fixation (Gallon et al., 1993). During summer, “*Ca.* Portiera” was an indicator for ambient temperature in seawater, consistent with our benthic mesocosm study at the North Sea during spring (Mensch et al., 2016). The genus “*Ca.* Portiera” (Bing et al., 2013) was discussed as error in the Greengenes database (Campbell et al., 2015). However, our data provide strong evidence that “*Ca.* Portiera” naturally occurs in marine habitats because it matched within the order *Oceanospirillales* reported to contain intracellular bacteria (Brenner et al., 2005a; Jensen et al., 2010). Additional iOTUs for ambient temperature belonged to the genera *Persicirhabdus* that appears nearly ubiquitous in the oceans (Yoon et al., 2008; Freitas et al., 2012), and *Roseibacillus* containing a strain isolated from a brown alga (Yoon et al., 2008). Together, these observations underline that several naturally occurring bacterial taxa in seawater as well as attached to macroalgal surfaces suffered from warming in this study.

In particular during spring and summer, iOTUs for increased temperature included bacteria that exhibit antifouling activity against macrofoulers, and that might represent potential pathogenic or parasitic bacteria. However, no evidence was found for potential pathogens at increased temperature during winter, possibly because anti-microfouling defense activity by *F. vesiculosus* was higher during the winter experiment under warming (see related data by Raddatz et al., 2017), and field observations on Baltic *F. vesiculosus* reported prokaryotic fouling to be lowest during winter months (Rickert et al., 2016a). Nevertheless, during winter, iOTUs that were favored by warming contained genera playing roles in fouling under high temperature and degradation/decomposition processes, like *Leadbetterella* (Weon et al., 2005) or the flavobacterial genus *Formosa* known for strains with algal-associated degradation potential (Ivanova et al., 2004; Nedashkovskaya et al., 2006; Mann et al., 2013). Proteobacterial “*Ca.* Endobugula” bacteria are symbionts of marine bryozoan (Lim and Haygood, 2004; Lim-Fong et al., 2008), in turn implying that warming during winter supported bryozoan growth as detected by increased numbers of their bacterial symbionts. Further, *Umboniibacter* species living in marine animals are known to be involved in the digestion of plant material and showed favored growth at temperatures above 5°C (Romanenko et al., 2010), consistent with the temperature reached in the warming treatments during the winter experiment (5–15°C range at +T).

Some iOTUs for increased temperature were found in both habitat types during spring and summer, like the epsilonproteobacterial genus *Arcobacter* that also include animal pathogens, besides non pathogenic free-living species in various habitats (Brenner et al., 2005b; Miller et al., 2007). During summer, an interesting epibacterial iOTU for increased temperature was *W. poriferorum* (Lau et al., 2005) known for antifouling potential including inhibitory effects on marine biofilm-forming bacteria and larval settlement of macrofoulers (Dash et al., 2009, 2011). That in turn could be important features on the surface of *F. vesiculosus* to stabilize its microbiota under high fouling pressure due to warming in summer. Some iOTUs for increased temperature belonged to groups containing single species associated with diseases such as pathogenic mycobacteria (uncl. *Mycobacterium*) known to be distributed also in seawater often causing infections of humans and animals (Dufour, 2004), and *Acholeplasma* within the class *Mollicutes* that include surface-associated pathogenic or saprotrophic species (characterized by lysotrophic nutrition, a process of chemoheterotrophic extracellular digestion involved in the processing of dead or decayed organic matter) (Krieg et al., 2010). The higher abundance of *Acholeplasma* under warming during summer might be connected with the temperature-related decay of *F. vesiculosus* in our benthocosm experiments (Graiff et al., 2015). As an overall observation, higher abundances of bacteria involved in intensified microfouling processes appear to correlate with a reduced Baltic *F. vesiculosus* biomass under warming in spring and summer (Graiff et al., 2015). Thus, our benthocosm studies suggest a strong connection between a thermally stressed macroalgal host (along with a reduced defense activity due to dying tissue and probably bacterial infections) with favored growth of bacteria associated with intensified microfouling.

## Conclusion

This study shows that increased temperature was a driving abiotic factor in reshaping bacterial communities on Baltic *F. vesiculosus* and in the surrounding seawater, and that elevated *p*CO_2_ had no direct impact, although in nature both climate phenomena – ocean acidification and global warming – are linked. Our detected temperature related iOTUs suggest that warming reduces commonly present and health-associated bacteria, but might also support growth of potential pathogenic bacteria, and favors bacteria that play a role in microfouling processes. This might lead to bacterial community restructuring with potentially severe consequences for the *F. vesiculosus* holobiont ultimately affecting the entire marine ecosystem.

## Data Availability Statement

The datasets generated for this study can be found in the NCBI Sequence Read Archive under accession number SRP075254 (https://www.ncbi.nlm.nih.gov/sra/?term=SRP075254) and at the PANGAEA^®^ data platform (https://doi.pangaea.de/10.1594/PANGAEA.842739).

## Author Contributions

BM designed the study, carried out the sampling and sample processing, analyzed and interpreted the data, and wrote the manuscript. SN performed the statistical analyses on the sequencing data. SK sequenced the amplicon samples. RS and MW designed the experiments, contributed the comments on the manuscript, founded, and supervised the work.

## Conflict of Interest

SN was employed by the company omics2view.consulting GbR, Kiel, Germany.

The remaining authors declare that the research was conducted in the absence of any commercial or financial relationships that could be construed as a potential conflict of interest.

## Footnotes

1 https://doi.pangaea.de/10.1594/PANGAEA.842739
