# Supplementary material for: Warming, but Not Acidification, Restructures Epibacterial Communities of the Baltic Macroalga Fucus vesiculosus With Seasonal Variability
Source: Front Microbiol. 2020 Jun 26;11:1471. doi: 10.3389/fmicb.2020.01471 (PMC7333354; doi:10.3389/fmicb.2020.01471)
Supplement: Supplementary file 1 [file Data_Sheet_1.PDF]

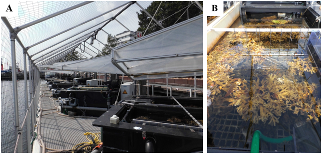

Fig. S1 Kiel Outdoor Benthocosms. A) The Kiel Outdoor Benthocosm facility on its floating platform located in the Kiel Fjord, Germany, with opened lids; and B) look into one of the subunits populated with *Fucus vesiculosus* (Photos by B. Mensch).
