# Supplementary material for: Warming, but Not Acidification, Restructures Epibacterial Communities of the Baltic Macroalga Fucus vesiculosus With Seasonal Variability
Source: Front Microbiol. 2020 Jun 26;11:1471. doi: 10.3389/fmicb.2020.01471 (PMC7333354; doi:10.3389/fmicb.2020.01471)
Supplement: Supplementary file 2 [file Data_Sheet_2.PDF]

A

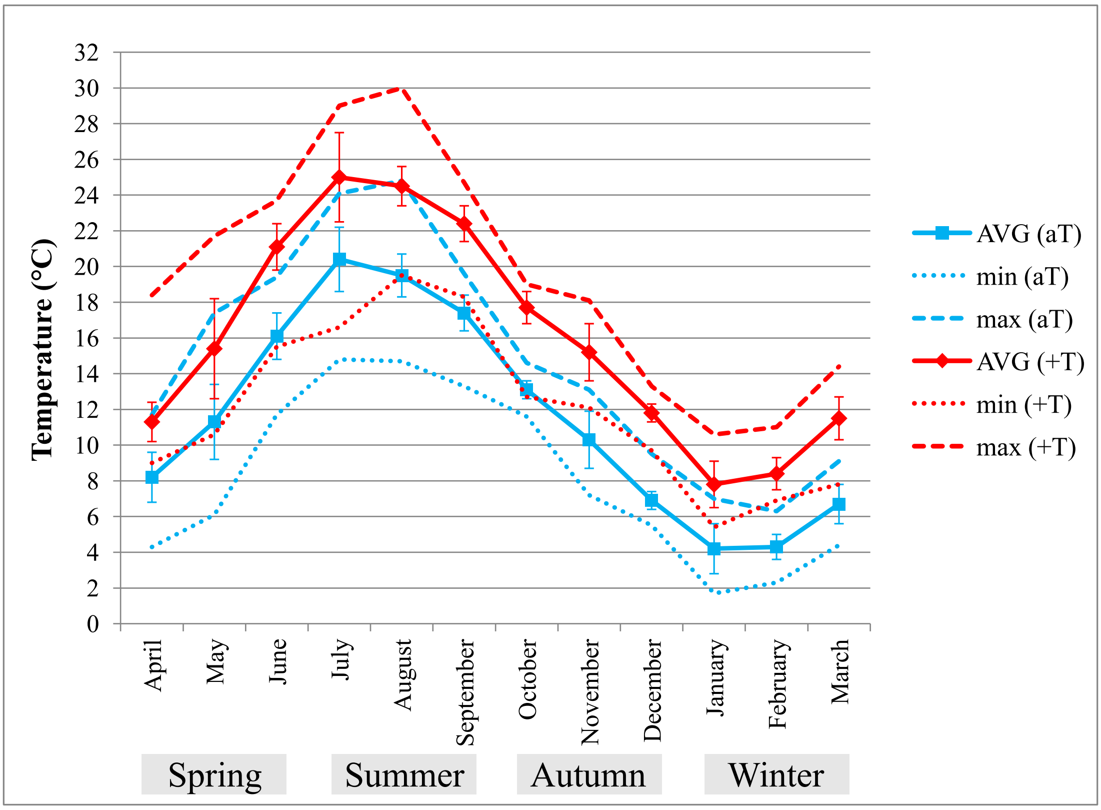

B

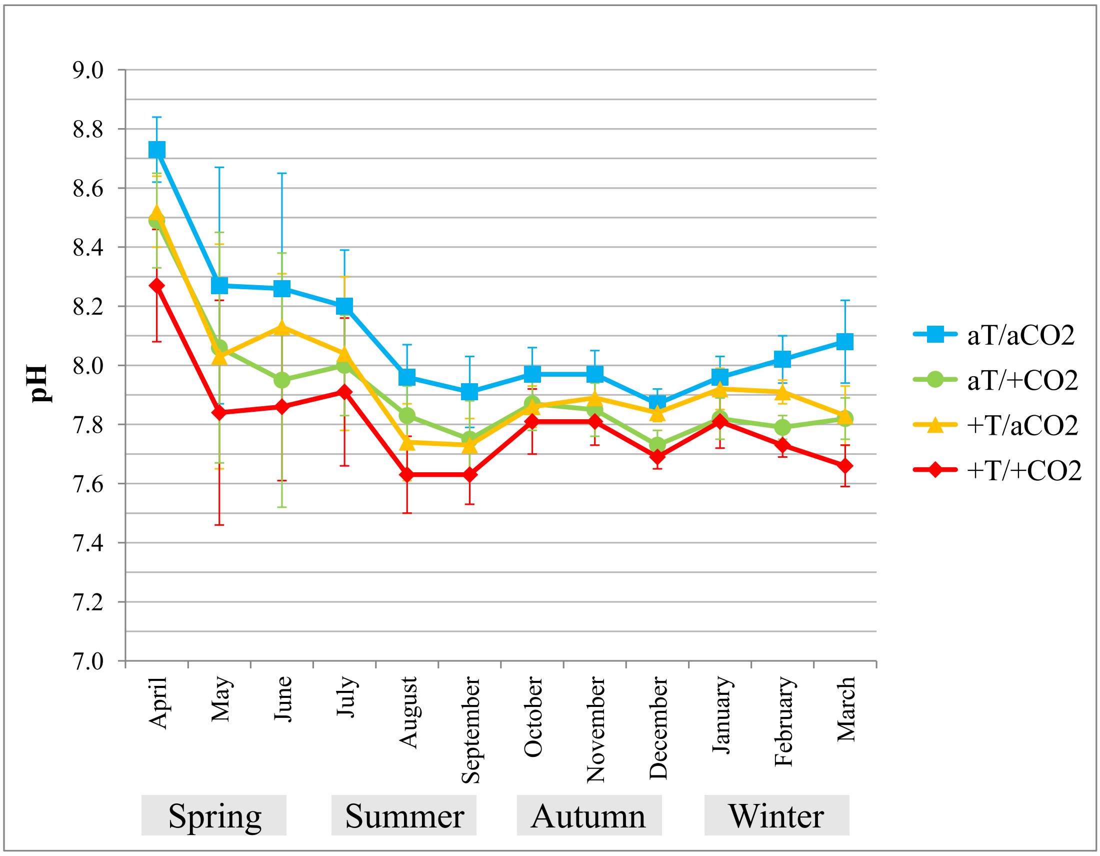

Fig. S2 Temperature and pH curves. A) Average (solid) with standard deviation, minimum (dotted) and maximum (dashed lines) temperature values for ambient (aT) and increased (+T) temperature treatments (each $n = 6$) in the benthocosms in every month. B) Average seawater pH (NBS scale) with standard deviation per treatment (four different conditions (see "Materials and Methods"; T, temperature; CO_2, pCO_2; a, ambient; +, increased), each $n = 3$). Data based on Graiff et al. (2015).
