# Supplementary material for: Warming, but Not Acidification, Restructures Epibacterial Communities of the Baltic Macroalga Fucus vesiculosus With Seasonal Variability
Source: Front Microbiol. 2020 Jun 26;11:1471. doi: 10.3389/fmicb.2020.01471 (PMC7333354; doi:10.3389/fmicb.2020.01471)
Supplement: Supplementary file 3 [file Data_Sheet_3.PDF]

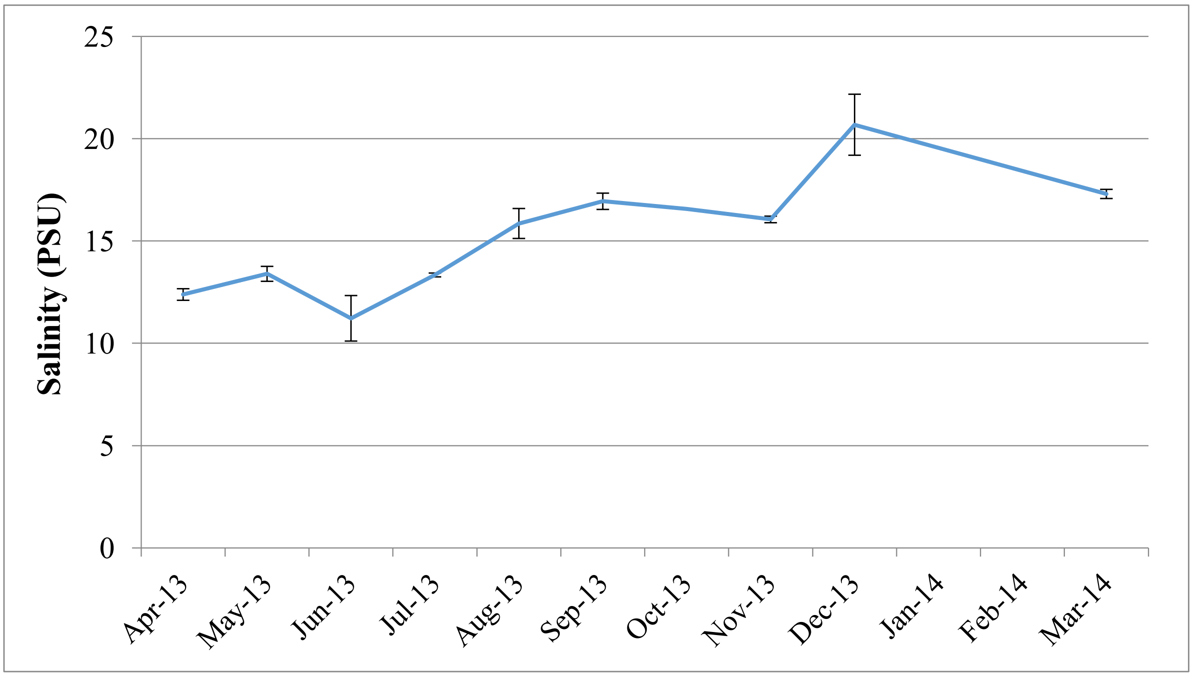

Fig. S3 Salinity curve. Monthly mean of the salinity (PSU, practical salinity units) measured in the Kiel Fjord, Germany, during April 2013 to March 2014.
