# Supplementary material for: Warming, but Not Acidification, Restructures Epibacterial Communities of the Baltic Macroalga Fucus vesiculosus With Seasonal Variability
Source: Front Microbiol. 2020 Jun 26;11:1471. doi: 10.3389/fmicb.2020.01471 (PMC7333354; doi:10.3389/fmicb.2020.01471)
Supplement: Supplementary file 4 [file Data_Sheet_4.PDF]

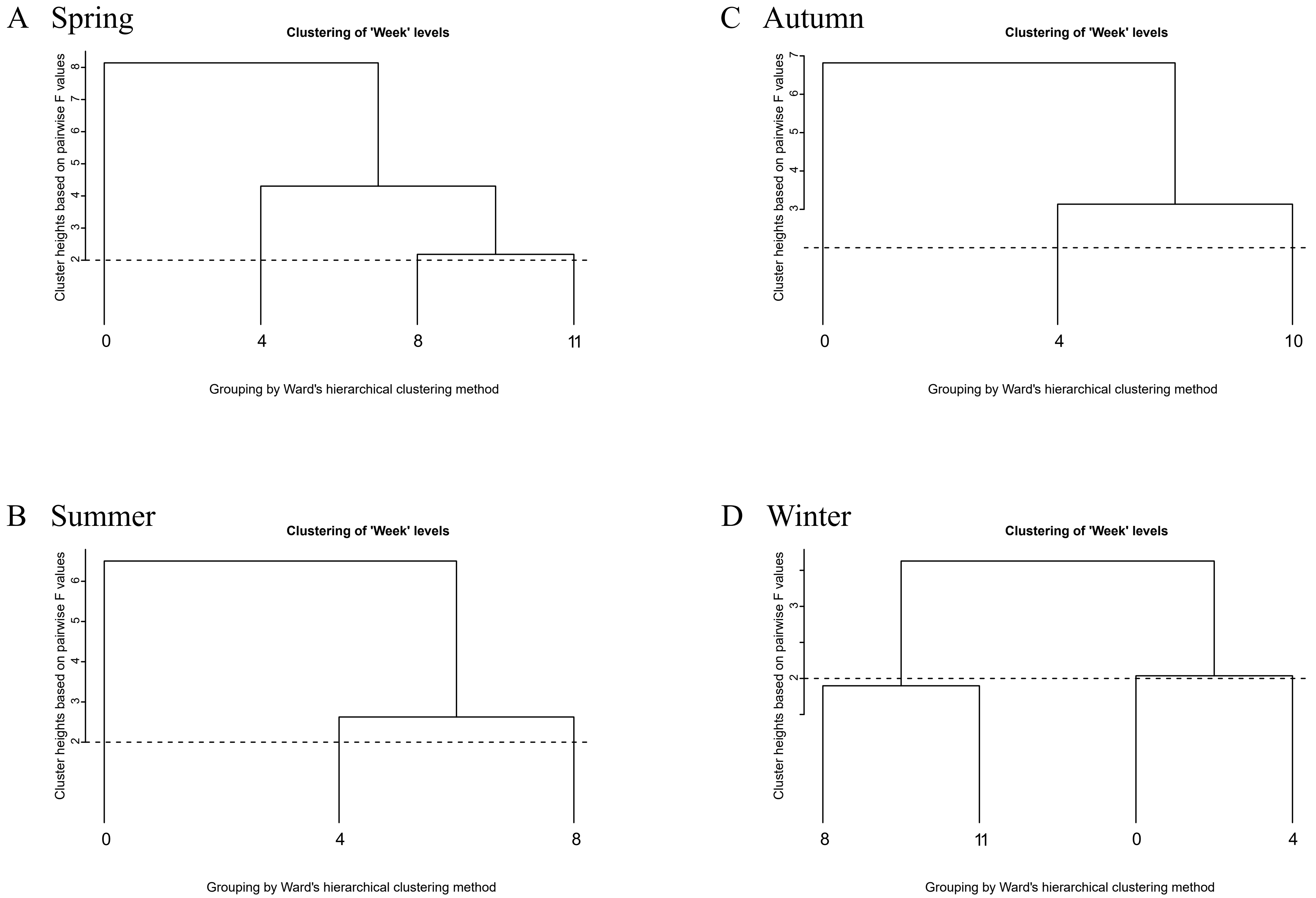

Fig. S4 Week-wise evaluation of community patterns of *Fucus vesiculosus* biofilm samples. Clustering of Week levels for A) spring, B) summer, C) autumn and D) winter. Grouping by Ward's hierarchical clustering method (for Weeks 0, 4, 8, 10/11) with cluster heights based on pairwise F values. F ≥ 2 indicates a threshold (dotted line) with bacterial communities differing markedly between weeks.
