# Supplementary material for: Warming, but Not Acidification, Restructures Epibacterial Communities of the Baltic Macroalga Fucus vesiculosus With Seasonal Variability
Source: Front Microbiol. 2020 Jun 26;11:1471. doi: 10.3389/fmicb.2020.01471 (PMC7333354; doi:10.3389/fmicb.2020.01471)
Supplement: Supplementary file 5 [file Data_Sheet_5.PDF]

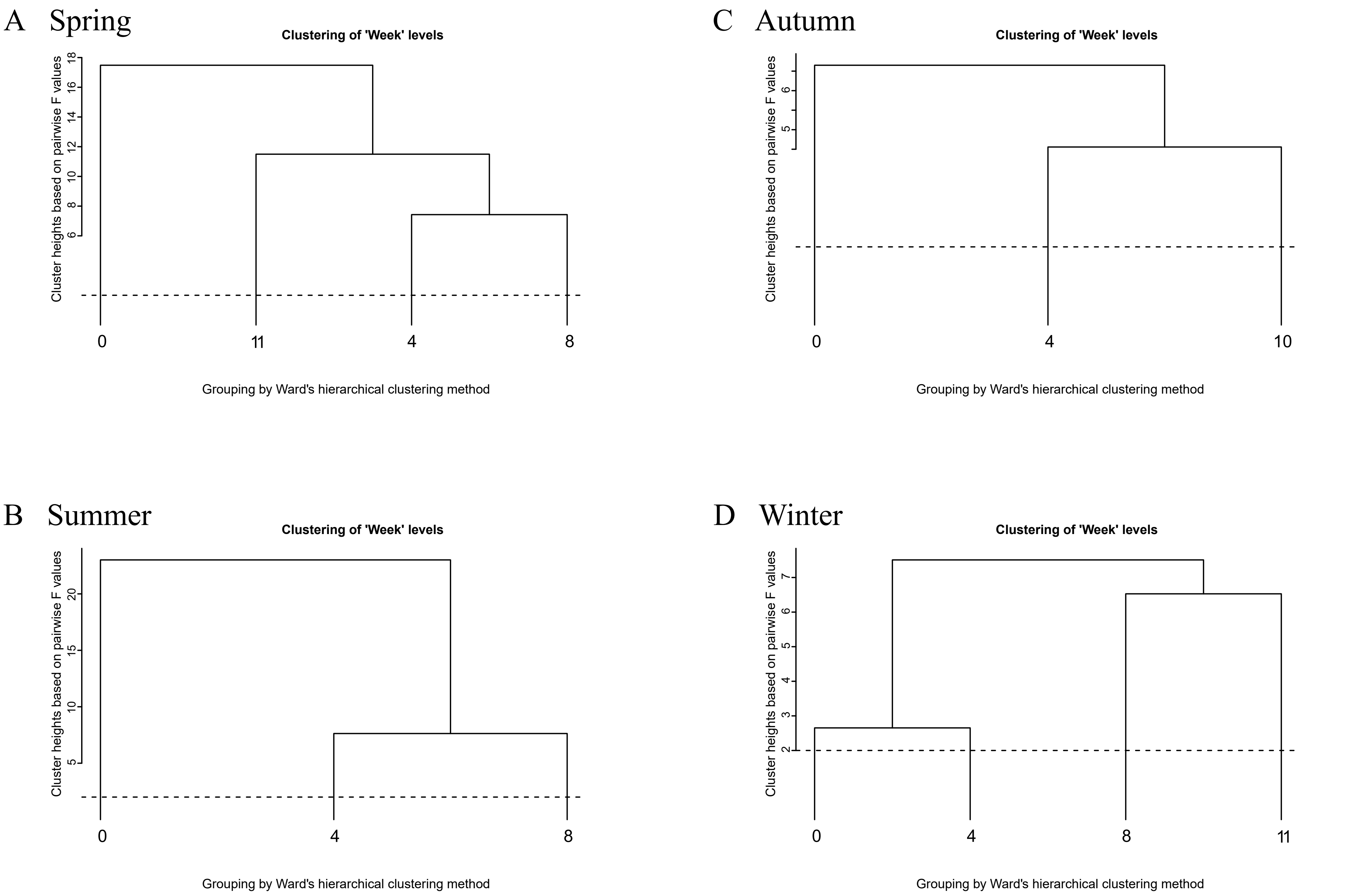

Fig. S5 Week-wise evaluation of community patterns in seawater samples. Clustering of Week levels for A) spring, B) summer, C) autumn and D) winter. Grouping by Ward's hierarchical clustering method (for Weeks 0, 4, 8, 10/11) with cluster heights based on pairwise F values. $F \geq 2$ indicates a threshold (dotted line) with bacterial communities differing markedly between weeks.
