# Supplementary material for: Warming, but Not Acidification, Restructures Epibacterial Communities of the Baltic Macroalga Fucus vesiculosus With Seasonal Variability
Source: Front Microbiol. 2020 Jun 26;11:1471. doi: 10.3389/fmicb.2020.01471 (PMC7333354; doi:10.3389/fmicb.2020.01471)
Supplement: Supplementary file 6 [file Data_Sheet_6.PDF]

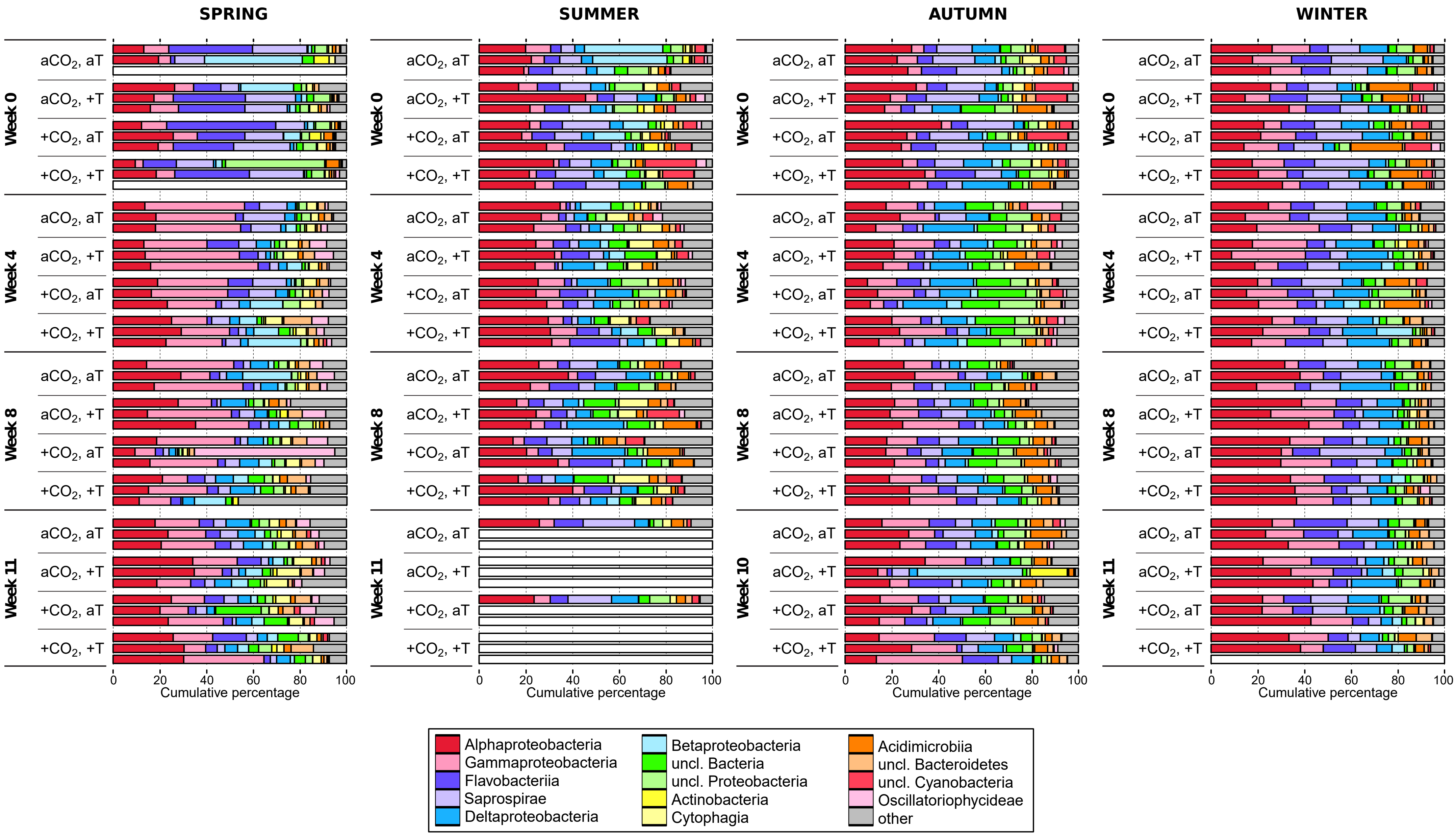

Fig. S6 Relative abundances of the most abundant epibacterial classes on *Fucus vesiculosus*. Epibacterial community composition is presented for each sample ($n = 3$ per treatment) after 0, 4, 8 and 10/11 weeks of treatment during spring, summer, autumn and winter, respectively, in the Kiel Benthocosms at four different conditions (see "Materials and Methods"; T, temperature; CO_2, pCO_2; a, ambient; +, increased). Analysis is based on bacterial 16S (V1-V2) MiSeq amplicon sequencing. Relative abundances of bacterial classes are given in cumulative percentage. Low abundant classes (≤1%) were summarized as "other". White bars indicate missing samples or sequence data (e.g., during summer in week 11 missing samples due to decayed *F. vesiculosus*).
