# Supplementary material for: Warming, but Not Acidification, Restructures Epibacterial Communities of the Baltic Macroalga Fucus vesiculosus With Seasonal Variability
Source: Front Microbiol. 2020 Jun 26;11:1471. doi: 10.3389/fmicb.2020.01471 (PMC7333354; doi:10.3389/fmicb.2020.01471)
Supplement: Supplementary file 8 [file Data_Sheet_8.PDF]

Tab. S1 Experimental design. Metadata table describing the experimental design including the factors Season (4 levels: Spring CE1, Summer CE2, Autumn CE3, Winter CE4), Type (2 levels: Biofilm_Fucus and Water), Week (4 levels: weeks 0, 4, 8, 11), Temp (2 levels: 0 = ambient T and +5 = +T) and *p*CO2 (2 levels: 0 = ambient CO2 and +600 = +CO2). CE, Core Experiment.

Sample_ID	Season	Sample_Unit	Exp_Unit	Type	Week	Temp	CO2
CE1_001	Spring	CE1_A1_W	A1	Water	CE1_00	+5	+600
CE1_005	Spring	CE1_C1_W	C1	Water	CE1_00	+5	+600
CE1_009	Spring	CE1_E1_W	E1	Water	CE1_00	+5	+600
CE1_013	Spring	CE1_A1_B	A1	Biofilm_Fucus	CE1_00	+5	+600
CE1_017	Spring	CE1_C1_B	C1	Biofilm_Fucus	CE1_00	+5	+600
CE1_021	Spring	CE1_E1_B	E1	Biofilm_Fucus	CE1_00	+5	+600
CE1_002	Spring	CE1_A2_W	A2	Water	CE1_00	+5	0
CE1_006	Spring	CE1_C2_W	C2	Water	CE1_00	+5	0
CE1_010	Spring	CE1_E2_W	E2	Water	CE1_00	+5	0
CE1_014	Spring	CE1_A2_B	A2	Biofilm_Fucus	CE1_00	+5	0
CE1_018	Spring	CE1_C2_B	C2	Biofilm_Fucus	CE1_00	+5	0
CE1_022	Spring	CE1_E2_B	E2	Biofilm_Fucus	CE1_00	+5	0
CE1_003	Spring	CE1_B1_W	B1	Water	CE1_00	0	+600
CE1_007	Spring	CE1_D1_W	D1	Water	CE1_00	0	+600
CE1_011	Spring	CE1_F1_W	F1	Water	CE1_00	0	+600
CE1_015	Spring	CE1_B1_B	B1	Biofilm_Fucus	CE1_00	0	+600
CE1_019	Spring	CE1_D1_B	D1	Biofilm_Fucus	CE1_00	0	+600
CE1_023	Spring	CE1_F1_B	F1	Biofilm_Fucus	CE1_00	0	+600
CE1_004	Spring	CE1_B2_W	B2	Water	CE1_00	0	0
CE1_008	Spring	CE1_D2_W	D2	Water	CE1_00	0	0
CE1_012	Spring	CE1_F2_W	F2	Water	CE1_00	0	0
CE1_016	Spring	CE1_B2_B	B2	Biofilm_Fucus	CE1_00	0	0
CE1_020	Spring	CE1_D2_B	D2	Biofilm_Fucus	CE1_00	0	0
CE1_024	Spring	CE1_F2_B	F2	Biofilm_Fucus	CE1_00	0	0
CE1_025	Spring	CE1_A1_W	A1	Water	CE1_04	+5	+600
CE1_029	Spring	CE1_C1_W	C1	Water	CE1_04	+5	+600
CE1_033	Spring	CE1_E1_W	E1	Water	CE1_04	+5	+600
CE1_037	Spring	CE1_A1_B	A1	Biofilm_Fucus	CE1_04	+5	+600
CE1_041	Spring	CE1_C1_B	C1	Biofilm_Fucus	CE1_04	+5	+600
CE1_045	Spring	CE1_E1_B	E1	Biofilm_Fucus	CE1_04	+5	+600
CE1_026	Spring	CE1_A2_W	A2	Water	CE1_04	+5	0
CE1_030	Spring	CE1_C2_W	C2	Water	CE1_04	+5	0
CE1_034	Spring	CE1_E2_W	E2	Water	CE1_04	+5	0
CE1_038	Spring	CE1_A2_B	A2	Biofilm_Fucus	CE1_04	+5	0
CE1_042	Spring	CE1_C2_B	C2	Biofilm_Fucus	CE1_04	+5	0
CE1_046	Spring	CE1_E2_B	E2	Biofilm_Fucus	CE1_04	+5	0
CE1_027	Spring	CE1_B1_W	B1	Water	CE1_04	0	+600
CE1_031	Spring	CE1_D1_W	D1	Water	CE1_04	0	+600
CE1_035	Spring	CE1_F1_W	F1	Water	CE1_04	0	+600
CE1_039	Spring	CE1_B1_B	B1	Biofilm_Fucus	CE1_04	0	+600
CE1_043	Spring	CE1_D1_B	D1	Biofilm_Fucus	CE1_04	0	+600
CE1_047	Spring	CE1_F1_B	F1	Biofilm_Fucus	CE1_04	0	+600
CE1_028	Spring	CE1_B2_W	B2	Water	CE1_04	0	0
CE1_032	Spring	CE1_D2_W	D2	Water	CE1_04	0	0
CE1_036	Spring	CE1_F2_W	F2	Water	CE1_04	0	0
CE1_040	Spring	CE1_B2_B	B2	Biofilm_Fucus	CE1_04	0	0
CE1_044	Spring	CE1_D2_B	D2	Biofilm_Fucus	CE1_04	0	0
CE1_048	Spring	CE1_F2_B	F2	Biofilm_Fucus	CE1_04	0	0
CE1_049	Spring	CE1_A1_W	A1	Water	CE1_08	+5	+600
CE1_053	Spring	CE1_C1_W	C1	Water	CE1_08	+5	+600
CE1_057	Spring	CE1_E1_W	E1	Water	CE1_08	+5	+600
CE1_061	Spring	CE1_A1_B	A1	Biofilm_Fucus	CE1_08	+5	+600
CE1_065	Spring	CE1_C1_B	C1	Biofilm_Fucus	CE1_08	+5	+600
CE1_069	Spring	CE1_E1_B	E1	Biofilm_Fucus	CE1_08	+5	+600
CE1_050	Spring	CE1_A2_W	A2	Water	CE1_08	+5	0
CE1_054	Spring	CE1_C2_W	C2	Water	CE1_08	+5	0
CE1_058	Spring	CE1_E2_W	E2	Water	CE1_08	+5	0
CE1_062	Spring	CE1_A2_B	A2	Biofilm_Fucus	CE1_08	+5	0
CE1_066	Spring	CE1_C2_B	C2	Biofilm_Fucus	CE1_08	+5	0
CE1_070	Spring	CE1_E2_B	E2	Biofilm_Fucus	CE1_08	+5	0
CE1_051	Spring	CE1_B1_W	B1	Water	CE1_08	0	+600
CE1_055	Spring	CE1_D1_W	D1	Water	CE1_08	0	+600
CE1_059	Spring	CE1_F1_W	F1	Water	CE1_08	0	+600
CE1_063	Spring	CE1_B1_B	B1	Biofilm_Fucus	CE1_08	0	+600
CE1_067	Spring	CE1_D1_B	D1	Biofilm_Fucus	CE1_08	0	+600
CE1_071	Spring	CE1_F1_B	F1	Biofilm_Fucus	CE1_08	0	+600
CE1_052	Spring	CE1_B2_W	B2	Water	CE1_08	0	0
CE1_056	Spring	CE1_D2_W	D2	Water	CE1_08	0	0
CE1_060	Spring	CE1_F2_W	F2	Water	CE1_08	0	0
CE1_064	Spring	CE1_B2_B	B2	Biofilm_Fucus	CE1_08	0	0
CE1_068	Spring	CE1_D2_B	D2	Biofilm_Fucus	CE1_08	0	0
CE1_072	Spring	CE1_F2_B	F2	Biofilm_Fucus	CE1_08	0	0
CE1_085	Spring	CE1_A1_W	A1	Water	CE1_11	+5	+600
CE1_089	Spring	CE1_C1_W	C1	Water	CE1_11	+5	+600
CE1_093	Spring	CE1_E1_W	E1	Water	CE1_11	+5	+600
CE1_097	Spring	CE1_A1_B	A1	Biofilm_Fucus	CE1_11	+5	+600
CE1_101	Spring	CE1_C1_B	C1	Biofilm_Fucus	CE1_11	+5	+600
CE1_105	Spring	CE1_E1_B	E1	Biofilm_Fucus	CE1_11	+5	+600
CE1_086	Spring	CE1_A2_W	A2	Water	CE1_11	+5	0
CE1_090	Spring	CE1_C2_W	C2	Water	CE1_11	+5	0
CE1_094	Spring	CE1_E2_W	E2	Water	CE1_11	+5	0
CE1_098	Spring	CE1_A2_B	A2	Biofilm_Fucus	CE1_11	+5	0
CE1_102	Spring	CE1_C2_B	C2	Biofilm_Fucus	CE1_11	+5	0
CE1_106	Spring	CE1_E2_B	E2	Biofilm_Fucus	CE1_11	+5	0
CE1_087	Spring	CE1_B1_W	B1	Water	CE1_11	0	+600
CE1_091	Spring	CE1_D1_W	D1	Water	CE1_11	0	+600
CE1_095	Spring	CE1_F1_W	F1	Water	CE1_11	0	+600
CE1_099	Spring	CE1_B1_B	B1	Biofilm_Fucus	CE1_11	0	+600
CE1_103	Spring	CE1_D1_B	D1	Biofilm_Fucus	CE1_11	0	+600
CE1_107	Spring	CE1_F1_B	F1	Biofilm_Fucus	CE1_11	0	+600
CE1_088	Spring	CE1_B2_W	B2	Water	CE1_11	0	0
CE1_092	Spring	CE1_D2_W	D2	Water	CE1_11	0	0
CE1_096	Spring	CE1_F2_W	F2	Water	CE1_11	0	0
CE1_100	Spring	CE1_B2_B	B2	Biofilm_Fucus	CE1_11	0	0
CE1_104	Spring	CE1_D2_B	D2	Biofilm_Fucus	CE1_11	0	0
CE1_108	Spring	CE1_F2_B	F2	Biofilm_Fucus	CE1_11	0	0
CE2_001	Summer	CE2_A1_W	A1	Water	CE2_00	+5	+600
CE2_005	Summer	CE2_C1_W	C1	Water	CE2_00	+5	+600
CE2_009	Summer	CE2_E1_W	E1	Water	CE2_00	+5	+600
CE2_013	Summer	CE2_A1_B	A1	Biofilm_Fucus	CE2_00	+5	+600
CE2_017	Summer	CE2_C1_B	C1	Biofilm_Fucus	CE2_00	+5	+600
CE2_021	Summer	CE2_E1_B	E1	Biofilm_Fucus	CE2_00	+5	+600
CE2_002	Summer	CE2_A2_W	A2	Water	CE2_00	+5	0
CE2_006	Summer	CE2_C2_W	C2	Water	CE2_00	+5	0
CE2_010	Summer	CE2_E2_W	E2	Water	CE2_00	+5	0
CE2_014	Summer	CE2_A2_B	A2	Biofilm_Fucus	CE2_00	+5	0
CE2_018	Summer	CE2_C2_B	C2	Biofilm_Fucus	CE2_00	+5	0
CE2_022	Summer	CE2_E2_B	E2	Biofilm_Fucus	CE2_00	+5	0
CE2_003	Summer	CE2_B1_W	B1	Water	CE2_00	0	+600
CE2_007	Summer	CE2_D1_W	D1	Water	CE2_00	0	+600
CE2_011	Summer	CE2_F1_W	F1	Water	CE2_00	0	+600
CE2_015	Summer	CE2_B1_B	B1	Biofilm_Fucus	CE2_00	0	+600
CE2_019	Summer	CE2_D1_B	D1	Biofilm_Fucus	CE2_00	0	+600
CE2_023	Summer	CE2_F1_B	F1	Biofilm_Fucus	CE2_00	0	+600
CE2_004	Summer	CE2_B2_W	B2	Water	CE2_00	0	0
CE2_008	Summer	CE2_D2_W	D2	Water	CE2_00	0	0
CE2_012	Summer	CE2_F2_W	F2	Water	CE2_00	0	0
CE2_016	Summer	CE2_B2_B	B2	Biofilm_Fucus	CE2_00	0	0
CE2_020	Summer	CE2_D2_B	D2	Biofilm_Fucus	CE2_00	0	0
CE2_024	Summer	CE2_F2_B	F2	Biofilm_Fucus	CE2_00	0	0
CE2_025	Summer	CE2_A1_W	A1	Water	CE2_04	+5	+600
CE2_029	Summer	CE2_C1_W	C1	Water	CE2_04	+5	+600
CE2_033	Summer	CE2_E1_W	E1	Water	CE2_04	+5	+600
CE2_037	Summer	CE2_A1_B	A1	Biofilm_Fucus	CE2_04	+5	+600
CE2_041	Summer	CE2_C1_B	C1	Biofilm_Fucus	CE2_04	+5	+600
CE2_045	Summer	CE2_E1_B	E1	Biofilm_Fucus	CE2_04	+5	+600
CE2_026	Summer	CE2_A2_W	A2	Water	CE2_04	+5	0
CE2_030	Summer	CE2_C2_W	C2	Water	CE2_04	+5	0
CE2_034	Summer	CE2_E2_W	E2	Water	CE2_04	+5	0
CE2_038	Summer	CE2_A2_B	A2	Biofilm_Fucus	CE2_04	+5	0
CE2_042	Summer	CE2_C2_B	C2	Biofilm_Fucus	CE2_04	+5	0
CE2_046	Summer	CE2_E2_B	E2	Biofilm_Fucus	CE2_04	+5	0
CE2_027	Summer	CE2_B1_W	B1	Water	CE2_04	0	+600
CE2_031	Summer	CE2_D1_W	D1	Water	CE2_04	0	+600
CE2_035	Summer	CE2_F1_W	F1	Water	CE2_04	0	+600
CE2_039	Summer	CE2_B1_B	B1	Biofilm_Fucus	CE2_04	0	+600
CE2_043	Summer	CE2_D1_B	D1	Biofilm_Fucus	CE2_04	0	+600
CE2_047	Summer	CE2_F1_B	F1	Biofilm_Fucus	CE2_04	0	+600
CE2_028	Summer	CE2_B2_W	B2	Water	CE2_04	0	0
CE2_032	Summer	CE2_D2_W	D2	Water	CE2_04	0	0
CE2_036	Summer	CE2_F2_W	F2	Water	CE2_04	0	0
CE2_040	Summer	CE2_B2_B	B2	Biofilm_Fucus	CE2_04	0	0
CE2_044	Summer	CE2_D2_B	D2	Biofilm_Fucus	CE2_04	0	0
CE2_048	Summer	CE2_F2_B	F2	Biofilm_Fucus	CE2_04	0	0
CE2_061	Summer	CE2_A1_W	A1	Water	CE2_08	+5	+600
CE2_065	Summer	CE2_C1_W	C1	Water	CE2_08	+5	+600
CE2_069	Summer	CE2_E1_W	E1	Water	CE2_08	+5	+600
CE2_073	Summer	CE2_A1_B	A1	Biofilm_Fucus	CE2_08	+5	+600
CE2_077	Summer	CE2_C1_B	C1	Biofilm_Fucus	CE2_08	+5	+600
CE2_081	Summer	CE2_E1_B	E1	Biofilm_Fucus	CE2_08	+5	+600
CE2_062	Summer	CE2_A2_W	A2	Water	CE2_08	+5	0
CE2_066	Summer	CE2_C2_W	C2	Water	CE2_08	+5	0
CE2_070	Summer	CE2_E2_W	E2	Water	CE2_08	+5	0
CE2_074	Summer	CE2_A2_B	A2	Biofilm_Fucus	CE2_08	+5	0
CE2_078	Summer	CE2_C2_B	C2	Biofilm_Fucus	CE2_08	+5	0
CE2_082	Summer	CE2_E2_B	E2	Biofilm_Fucus	CE2_08	+5	0
CE2_063	Summer	CE2_B1_W	B1	Water	CE2_08	0	+600
CE2_067	Summer	CE2_D1_W	D1	Water	CE2_08	0	+600
CE2_071	Summer	CE2_F1_W	F1	Water	CE2_08	0	+600
CE2_075	Summer	CE2_B1_B	B1	Biofilm_Fucus	CE2_08	0	+600
CE2_079	Summer	CE2_D1_B	D1	Biofilm_Fucus	CE2_08	0	+600
CE2_083	Summer	CE2_F1_B	F1	Biofilm_Fucus	CE2_08	0	+600
CE2_064	Summer	CE2_B2_W	B2	Water	CE2_08	0	0
CE2_068	Summer	CE2_D2_W	D2	Water	CE2_08	0	0
CE2_072	Summer	CE2_F2_W	F2	Water	CE2_08	0	0
CE2_076	Summer	CE2_B2_B	B2	Biofilm_Fucus	CE2_08	0	0
CE2_080	Summer	CE2_D2_B	D2	Biofilm_Fucus	CE2_08	0	0
CE2_084	Summer	CE2_F2_B	F2	Biofilm_Fucus	CE2_08	0	0
CE2_097	Summer	CE2_A1_W	A1	Water	CE2_11	+5	+600
CE2_101	Summer	CE2_C1_W	C1	Water	CE2_11	+5	+600
CE2_105	Summer	CE2_E1_W	E1	Water	CE2_11	+5	+600
CE2_098	Summer	CE2_A2_W	A2	Water	CE2_11	+5	0
CE2_102	Summer	CE2_C2_W	C2	Water	CE2_11	+5	0
CE2_106	Summer	CE2_E2_W	E2	Water	CE2_11	+5	0
CE2_099	Summer	CE2_B1_W	B1	Water	CE2_11	0	+600
CE2_103	Summer	CE2_D1_W	D1	Water	CE2_11	0	+600
CE2_107	Summer	CE2_F1_W	F1	Water	CE2_11	0	+600
CE2_109	Summer	CE2_B1_B	B1	Biofilm_Fucus	CE2_11	0	+600
CE2_111	Summer	CE2_D1_B	D1	Biofilm_Fucus	CE2_11	0	+600
CE2_113	Summer	CE2_F1_B	F1	Biofilm_Fucus	CE2_11	0	+600
CE2_100	Summer	CE2_B2_W	B2	Water	CE2_11	0	0
CE2_104	Summer	CE2_D2_W	D2	Water	CE2_11	0	0
CE2_108	Summer	CE2_F2_W	F2	Water	CE2_11	0	0
CE2_110	Summer	CE2_B2_B	B2	Biofilm_Fucus	CE2_11	0	0
CE2_112	Summer	CE2_D2_B	D2	Biofilm_Fucus	CE2_11	0	0
CE2_114	Summer	CE2_F2_B	F2	Biofilm_Fucus	CE2_11	0	0
CE3_001	Autumn	CE3_A1_W	A1	Water	CE3_00	+5	+600
CE3_005	Autumn	CE3_C1_W	C1	Water	CE3_00	+5	+600
CE3_009	Autumn	CE3_E1_W	E1	Water	CE3_00	+5	+600
CE3_013	Autumn	CE3_A1_B	A1	Biofilm_Fucus	CE3_00	+5	+600
CE3_017	Autumn	CE3_C1_B	C1	Biofilm_Fucus	CE3_00	+5	+600
CE3_021	Autumn	CE3_E1_B	E1	Biofilm_Fucus	CE3_00	+5	+600
CE3_002	Autumn	CE3_A2_W	A2	Water	CE3_00	+5	0
CE3_006	Autumn	CE3_C2_W	C2	Water	CE3_00	+5	0
CE3_010	Autumn	CE3_E2_W	E2	Water	CE3_00	+5	0
CE3_014	Autumn	CE3_A2_B	A2	Biofilm_Fucus	CE3_00	+5	0
CE3_018	Autumn	CE3_C2_B	C2	Biofilm_Fucus	CE3_00	+5	0
CE3_022	Autumn	CE3_E2_B	E2	Biofilm_Fucus	CE3_00	+5	0
CE3_003	Autumn	CE3_B1_W	B1	Water	CE3_00	0	+600
CE3_007	Autumn	CE3_D1_W	D1	Water	CE3_00	0	+600
CE3_011	Autumn	CE3_F1_W	F1	Water	CE3_00	0	+600
CE3_015	Autumn	CE3_B1_B	B1	Biofilm_Fucus	CE3_00	0	+600
CE3_019	Autumn	CE3_D1_B	D1	Biofilm_Fucus	CE3_00	0	+600
CE3_023	Autumn	CE3_F1_B	F1	Biofilm_Fucus	CE3_00	0	+600
CE3_004	Autumn	CE3_B2_W	B2	Water	CE3_00	0	0
CE3_008	Autumn	CE3_D2_W	D2	Water	CE3_00	0	0
CE3_012	Autumn	CE3_F2_W	F2	Water	CE3_00	0	0
CE3_016	Autumn	CE3_B2_B	B2	Biofilm_Fucus	CE3_00	0	0
CE3_020	Autumn	CE3_D2_B	D2	Biofilm_Fucus	CE3_00	0	0
CE3_024	Autumn	CE3_F2_B	F2	Biofilm_Fucus	CE3_00	0	0
CE3_025	Autumn	CE3_A1_W	A1	Water	CE3_04	+5	+600
CE3_029	Autumn	CE3_C1_W	C1	Water	CE3_04	+5	+600
CE3_033	Autumn	CE3_E1_W	E1	Water	CE3_04	+5	+600
CE3_037	Autumn	CE3_A1_B	A1	Biofilm_Fucus	CE3_04	+5	+600
CE3_041	Autumn	CE3_C1_B	C1	Biofilm_Fucus	CE3_04	+5	+600
CE3_045	Autumn	CE3_E1_B	E1	Biofilm_Fucus	CE3_04	+5	+600
CE3_026	Autumn	CE3_A2_W	A2	Water	CE3_04	+5	0
CE3_030	Autumn	CE3_C2_W	C2	Water	CE3_04	+5	0
CE3_034	Autumn	CE3_E2_W	E2	Water	CE3_04	+5	0
CE3_038	Autumn	CE3_A2_B	A2	Biofilm_Fucus	CE3_04	+5	0
CE3_042	Autumn	CE3_C2_B	C2	Biofilm_Fucus	CE3_04	+5	0
CE3_046	Autumn	CE3_E2_B	E2	Biofilm_Fucus	CE3_04	+5	0
CE3_027	Autumn	CE3_B1_W	B1	Water	CE3_04	0	+600
CE3_031	Autumn	CE3_D1_W	D1	Water	CE3_04	0	+600
CE3_035	Autumn	CE3_F1_W	F1	Water	CE3_04	0	+600
CE3_039	Autumn	CE3_B1_B	B1	Biofilm_Fucus	CE3_04	0	+600
CE3_043	Autumn	CE3_D1_B	D1	Biofilm_Fucus	CE3_04	0	+600
CE3_047	Autumn	CE3_F1_B	F1	Biofilm_Fucus	CE3_04	0	+600
CE3_028	Autumn	CE3_B2_W	B2	Water	CE3_04	0	0
CE3_032	Autumn	CE3_D2_W	D2	Water	CE3_04	0	0
CE3_036	Autumn	CE3_F2_W	F2	Water	CE3_04	0	0
CE3_040	Autumn	CE3_B2_B	B2	Biofilm_Fucus	CE3_04	0	0
CE3_044	Autumn	CE3_D2_B	D2	Biofilm_Fucus	CE3_04	0	0
CE3_048	Autumn	CE3_F2_B	F2	Biofilm_Fucus	CE3_04	0	0
CE3_061	Autumn	CE3_A1_B	A1	Biofilm_Fucus	CE3_08	+5	+600
CE3_065	Autumn	CE3_C1_B	C1	Biofilm_Fucus	CE3_08	+5	+600
CE3_069	Autumn	CE3_E1_B	E1	Biofilm_Fucus	CE3_08	+5	+600
CE3_062	Autumn	CE3_A2_B	A2	Biofilm_Fucus	CE3_08	+5	0
CE3_066	Autumn	CE3_C2_B	C2	Biofilm_Fucus	CE3_08	+5	0
CE3_070	Autumn	CE3_E2_B	E2	Biofilm_Fucus	CE3_08	+5	0
CE3_063	Autumn	CE3_B1_B	B1	Biofilm_Fucus	CE3_08	0	+600
CE3_067	Autumn	CE3_D1_B	D1	Biofilm_Fucus	CE3_08	0	+600
CE3_071	Autumn	CE3_F1_B	F1	Biofilm_Fucus	CE3_08	0	+600
CE3_064	Autumn	CE3_B2_B	B2	Biofilm_Fucus	CE3_08	0	0
CE3_068	Autumn	CE3_D2_B	D2	Biofilm_Fucus	CE3_08	0	0
CE3_072	Autumn	CE3_F2_B	F2	Biofilm_Fucus	CE3_08	0	0
CE3_085	Autumn	CE3_A1_W	A1	Water	CE3_11	+5	+600
CE3_089	Autumn	CE3_C1_W	C1	Water	CE3_11	+5	+600
CE3_093	Autumn	CE3_E1_W	E1	Water	CE3_11	+5	+600
CE3_097	Autumn	CE3_A1_B	A1	Biofilm_Fucus	CE3_11	+5	+600
CE3_101	Autumn	CE3_C1_B	C1	Biofilm_Fucus	CE3_11	+5	+600
CE3_105	Autumn	CE3_E1_B	E1	Biofilm_Fucus	CE3_11	+5	+600
CE3_086	Autumn	CE3_A2_W	A2	Water	CE3_11	+5	0
CE3_090	Autumn	CE3_C2_W	C2	Water	CE3_11	+5	0
CE3_094	Autumn	CE3_E2_W	E2	Water	CE3_11	+5	0
CE3_098	Autumn	CE3_A2_B	A2	Biofilm_Fucus	CE3_11	+5	0
CE3_102	Autumn	CE3_C2_B	C2	Biofilm_Fucus	CE3_11	+5	0
CE3_106	Autumn	CE3_E2_B	E2	Biofilm_Fucus	CE3_11	+5	0
CE3_087	Autumn	CE3_B1_W	B1	Water	CE3_11	0	+600
CE3_091	Autumn	CE3_D1_W	D1	Water	CE3_11	0	+600
CE3_095	Autumn	CE3_F1_W	F1	Water	CE3_11	0	+600
CE3_099	Autumn	CE3_B1_B	B1	Biofilm_Fucus	CE3_11	0	+600
CE3_103	Autumn	CE3_D1_B	D1	Biofilm_Fucus	CE3_11	0	+600
CE3_107	Autumn	CE3_F1_B	F1	Biofilm_Fucus	CE3_11	0	+600
CE3_088	Autumn	CE3_B2_W	B2	Water	CE3_11	0	0
CE3_092	Autumn	CE3_D2_W	D2	Water	CE3_11	0	0
CE3_096	Autumn	CE3_F2_W	F2	Water	CE3_11	0	0
CE3_100	Autumn	CE3_B2_B	B2	Biofilm_Fucus	CE3_11	0	0
CE3_104	Autumn	CE3_D2_B	D2	Biofilm_Fucus	CE3_11	0	0
CE3_108	Autumn	CE3_F2_B	F2	Biofilm_Fucus	CE3_11	0	0
CE4_001	Winter	CE4_A1_W	A1	Water	CE4_00	+5	+600
CE4_005	Winter	CE4_C1_W	C1	Water	CE4_00	+5	+600
CE4_009	Winter	CE4_E1_W	E1	Water	CE4_00	+5	+600
CE4_013	Winter	CE4_A1_B	A1	Biofilm_Fucus	CE4_00	+5	+600
CE4_017	Winter	CE4_C1_B	C1	Biofilm_Fucus	CE4_00	+5	+600
CE4_021	Winter	CE4_E1_B	E1	Biofilm_Fucus	CE4_00	+5	+600
CE4_002	Winter	CE4_A2_W	A2	Water	CE4_00	+5	0
CE4_006	Winter	CE4_C2_W	C2	Water	CE4_00	+5	0
CE4_010	Winter	CE4_E2_W	E2	Water	CE4_00	+5	0
CE4_014	Winter	CE4_A2_B	A2	Biofilm_Fucus	CE4_00	+5	0
CE4_018	Winter	CE4_C2_B	C2	Biofilm_Fucus	CE4_00	+5	0
CE4_022	Winter	CE4_E2_B	E2	Biofilm_Fucus	CE4_00	+5	0
CE4_003	Winter	CE4_B1_W	B1	Water	CE4_00	0	+600
CE4_007	Winter	CE4_D1_W	D1	Water	CE4_00	0	+600
CE4_011	Winter	CE4_F1_W	F1	Water	CE4_00	0	+600
CE4_015	Winter	CE4_B1_B	B1	Biofilm_Fucus	CE4_00	0	+600
CE4_019	Winter	CE4_D1_B	D1	Biofilm_Fucus	CE4_00	0	+600
CE4_023	Winter	CE4_F1_B	F1	Biofilm_Fucus	CE4_00	0	+600
CE4_004	Winter	CE4_B2_W	B2	Water	CE4_00	0	0
CE4_008	Winter	CE4_D2_W	D2	Water	CE4_00	0	0
CE4_012	Winter	CE4_F2_W	F2	Water	CE4_00	0	0
CE4_016	Winter	CE4_B2_B	B2	Biofilm_Fucus	CE4_00	0	0
CE4_020	Winter	CE4_D2_B	D2	Biofilm_Fucus	CE4_00	0	0
CE4_024	Winter	CE4_F2_B	F2	Biofilm_Fucus	CE4_00	0	0
CE4_025	Winter	CE4_A1_W	A1	Water	CE4_04	+5	+600
CE4_029	Winter	CE4_C1_W	C1	Water	CE4_04	+5	+600
CE4_033	Winter	CE4_E1_W	E1	Water	CE4_04	+5	+600
CE4_037	Winter	CE4_A1_B	A1	Biofilm_Fucus	CE4_04	+5	+600
CE4_041	Winter	CE4_C1_B	C1	Biofilm_Fucus	CE4_04	+5	+600
CE4_045	Winter	CE4_E1_B	E1	Biofilm_Fucus	CE4_04	+5	+600
CE4_026	Winter	CE4_A2_W	A2	Water	CE4_04	+5	0
CE4_030	Winter	CE4_C2_W	C2	Water	CE4_04	+5	0
CE4_034	Winter	CE4_E2_W	E2	Water	CE4_04	+5	0
CE4_038	Winter	CE4_A2_B	A2	Biofilm_Fucus	CE4_04	+5	0
CE4_042	Winter	CE4_C2_B	C2	Biofilm_Fucus	CE4_04	+5	0
CE4_046	Winter	CE4_E2_B	E2	Biofilm_Fucus	CE4_04	+5	0
CE4_027	Winter	CE4_B1_W	B1	Water	CE4_04	0	+600
CE4_031	Winter	CE4_D1_W	D1	Water	CE4_04	0	+600
CE4_035	Winter	CE4_F1_W	F1	Water	CE4_04	0	+600
CE4_039	Winter	CE4_B1_B	B1	Biofilm_Fucus	CE4_04	0	+600
CE4_043	Winter	CE4_D1_B	D1	Biofilm_Fucus	CE4_04	0	+600
CE4_047	Winter	CE4_F1_B	F1	Biofilm_Fucus	CE4_04	0	+600
CE4_028	Winter	CE4_B2_W	B2	Water	CE4_04	0	0
CE4_032	Winter	CE4_D2_W	D2	Water	CE4_04	0	0
CE4_036	Winter	CE4_F2_W	F2	Water	CE4_04	0	0
CE4_040	Winter	CE4_B2_B	B2	Biofilm_Fucus	CE4_04	0	0
CE4_044	Winter	CE4_D2_B	D2	Biofilm_Fucus	CE4_04	0	0
CE4_048	Winter	CE4_F2_B	F2	Biofilm_Fucus	CE4_04	0	0
CE4_061	Winter	CE4_A1_W	A1	Water	CE4_08	+5	+600
CE4_065	Winter	CE4_C1_W	C1	Water	CE4_08	+5	+600
CE4_069	Winter	CE4_E1_W	E1	Water	CE4_08	+5	+600
CE4_073	Winter	CE4_A1_B	A1	Biofilm_Fucus	CE4_08	+5	+600
CE4_077	Winter	CE4_C1_B	C1	Biofilm_Fucus	CE4_08	+5	+600
CE4_081	Winter	CE4_E1_B	E1	Biofilm_Fucus	CE4_08	+5	+600
CE4_062	Winter	CE4_A2_W	A2	Water	CE4_08	+5	0
CE4_066	Winter	CE4_C2_W	C2	Water	CE4_08	+5	0
CE4_070	Winter	CE4_E2_W	E2	Water	CE4_08	+5	0
CE4_074	Winter	CE4_A2_B	A2	Biofilm_Fucus	CE4_08	+5	0
CE4_078	Winter	CE4_C2_B	C2	Biofilm_Fucus	CE4_08	+5	0
CE4_082	Winter	CE4_E2_B	E2	Biofilm_Fucus	CE4_08	+5	0
CE4_063	Winter	CE4_B1_W	B1	Water	CE4_08	0	+600
CE4_067	Winter	CE4_D1_W	D1	Water	CE4_08	0	+600
CE4_071	Winter	CE4_F1_W	F1	Water	CE4_08	0	+600
CE4_075	Winter	CE4_B1_B	B1	Biofilm_Fucus	CE4_08	0	+600
CE4_079	Winter	CE4_D1_B	D1	Biofilm_Fucus	CE4_08	0	+600
CE4_083	Winter	CE4_F1_B	F1	Biofilm_Fucus	CE4_08	0	+600
CE4_064	Winter	CE4_B2_W	B2	Water	CE4_08	0	0
CE4_068	Winter	CE4_D2_W	D2	Water	CE4_08	0	0
CE4_072	Winter	CE4_F2_W	F2	Water	CE4_08	0	0
CE4_076	Winter	CE4_B2_B	B2	Biofilm_Fucus	CE4_08	0	0
CE4_080	Winter	CE4_D2_B	D2	Biofilm_Fucus	CE4_08	0	0
CE4_084	Winter	CE4_F2_B	F2	Biofilm_Fucus	CE4_08	0	0
CE4_097	Winter	CE4_A1_W	A1	Water	CE4_11	+5	+600
CE4_101	Winter	CE4_C1_W	C1	Water	CE4_11	+5	+600
CE4_105	Winter	CE4_E1_W	E1	Water	CE4_11	+5	+600
CE4_109	Winter	CE4_A1_B	A1	Biofilm_Fucus	CE4_11	+5	+600
CE4_113	Winter	CE4_C1_B	C1	Biofilm_Fucus	CE4_11	+5	+600
CE4_117	Winter	CE4_E1_B	E1	Biofilm_Fucus	CE4_11	+5	+600
CE4_098	Winter	CE4_A2_W	A2	Water	CE4_11	+5	0
CE4_102	Winter	CE4_C2_W	C2	Water	CE4_11	+5	0
CE4_106	Winter	CE4_E2_W	E2	Water	CE4_11	+5	0
CE4_110	Winter	CE4_A2_B	A2	Biofilm_Fucus	CE4_11	+5	0
CE4_114	Winter	CE4_C2_B	C2	Biofilm_Fucus	CE4_11	+5	0
CE4_118	Winter	CE4_E2_B	E2	Biofilm_Fucus	CE4_11	+5	0
CE4_099	Winter	CE4_B1_W	B1	Water	CE4_11	0	+600
CE4_103	Winter	CE4_D1_W	D1	Water	CE4_11	0	+600
CE4_107	Winter	CE4_F1_W	F1	Water	CE4_11	0	+600
CE4_111	Winter	CE4_B1_B	B1	Biofilm_Fucus	CE4_11	0	+600
CE4_115	Winter	CE4_D1_B	D1	Biofilm_Fucus	CE4_11	0	+600
CE4_119	Winter	CE4_F1_B	F1	Biofilm_Fucus	CE4_11	0	+600
CE4_100	Winter	CE4_B2_W	B2	Water	CE4_11	0	0
CE4_104	Winter	CE4_D2_W	D2	Water	CE4_11	0	0
CE4_108	Winter	CE4_F2_W	F2	Water	CE4_11	0	0
CE4_112	Winter	CE4_B2_B	B2	Biofilm_Fucus	CE4_11	0	0
CE4_116	Winter	CE4_D2_B	D2	Biofilm_Fucus	CE4_11	0	0
CE4_120	Winter	CE4_F2_B	F2	Biofilm_Fucus	CE4_11	0	0
