# Supplementary material for: Warming, but Not Acidification, Restructures Epibacterial Communities of the Baltic Macroalga Fucus vesiculosus With Seasonal Variability
Source: Front Microbiol. 2020 Jun 26;11:1471. doi: 10.3389/fmicb.2020.01471 (PMC7333354; doi:10.3389/fmicb.2020.01471)
Supplement: Supplementary file 9 [file Data_Sheet_9.PDF]

Tab. S2 Taxonomy of OTUs correlated within OTU association network. Taxonomy of the 42 bacterial OTUs forming the OTU association network (see **Fig. 5**) sorted by OTU number. Proteo., Proteobacteria.

OTU	Phylum	Class	Order	Family	Genus	Species
**1**	Proteobacteria	Alphaproteo.	Rickettsiales	Pelagibacteraceae	Pelagibacter	*Pelagibacter ubique*
**2**	Bacteroidetes	Flavobacteriia	Flavobacteriales	Cryomorphaceae	uncl. Cryomorphaceae	uncl. Cryomorphaceae
**3**	Proteobacteria	Alphaproteo.	Rhodobacterales	Rhodobacteraceae	Octadecabacter	uncl. Octadecabacter
**4**	Proteobacteria	Alphaproteo.	Rickettsiales	Pelagibacteraceae	uncl. Pelagibacteraceae	uncl. Pelagibacteraceae
**5**	Proteobacteria	Gammaproteo.	Oceanospirillales	Halomonadaceae	Candidatus Portiera	uncl. Candidatus Portiera
**6**	Proteobacteria	Alphaproteo.	Rickettsiales	Pelagibacteraceae	uncl. Pelagibacteraceae	uncl. Pelagibacteraceae
**7**	Proteobacteria	Alphaproteo.	Rhizobiales	Bradyrhizobiaceae	uncl. Bradyrhizobiaceae	uncl. Bradyrhizobiaceae
**8**	Proteobacteria	Betaproteo.	Methylophilales	Methylophilaceae	uncl. Methylophilaceae	uncl. Methylophilaceae
**9**	Bacteroidetes	Flavobacteriia	Flavobacteriales	Flavobacteriaceae	Flavobacterium	uncl. Flavobacterium
**10**	Proteobacteria	Betaproteo.	Burkholderiales	Comamonadaceae	uncl. Comamonadaceae	uncl. Comamonadaceae
**11**	Proteobacteria	Gammaproteo.	Alteromonadales	Alteromonadaceae	Glaciecola	uncl. Glaciecola
**12**	Proteobacteria	Alphaproteo.	Rhodobacterales	Rhodobacteraceae	uncl. Rhodobacteraceae	uncl. Rhodobacteraceae
**13**	Proteobacteria	Alphaproteo.	Rickettsiales	Pelagibacteraceae	uncl. Pelagibacteraceae	uncl. Pelagibacteraceae
**14**	Proteobacteria	Alphaproteo.	Rickettsiales	Pelagibacteraceae	uncl. Pelagibacteraceae	uncl. Pelagibacteraceae
**15**	Actinobacteria	Actinobacteria	Actinomycetales	uncl. Actinomycetales	uncl. Actinomycetales	uncl. Actinomycetales
**16**	Bacteroidetes	[Saprospirae]	[Saprospirales]	Saprospiraceae	uncl. Saprospiraceae	uncl. Saprospiraceae
**17**	Proteobacteria	Alphaproteo.	Sphingomonadales	Erythrobacteraceae	Erythrobacter	uncl. Erythrobacter
**18**	Proteobacteria	Deltaproteo.	Myxococcales	Polyangiaceae	uncl. Polyangiaceae	uncl. Polyangiaceae
**21**	Cyanobacteria	Oscillatoriophycideae	Chroococcales	uncl. Chroococcales	uncl. Chroococcales	uncl. Chroococcales
**22**	Proteobacteria	uncl. Proteo.	uncl. Proteo.	uncl. Proteo.	uncl. Proteo.	uncl. Proteo.
**23**	Proteobacteria	uncl. Proteo.	uncl. Proteo.	uncl. Proteo.	uncl. Proteo.	uncl. Proteo.
**24**	Tenericutes	Mollicutes	Acholeplasmatales	Acholeplasmataceae	Acholeplasma	uncl. Acholeplasma
**25**	Proteobacteria	Gammaproteo.	Oceanospirillales	SUP05	uncl. SUP05	uncl. SUP05
**28**	Proteobacteria	Alphaproteo.	Rhodobacterales	Rhodobacteraceae	uncl. Rhodobacteraceae	uncl. Rhodobacteraceae
**29**	Proteobacteria	Betaproteo.	Methylophilales	Methylophilaceae	uncl. Methylophilaceae	uncl. Methylophilaceae
**30**	Proteobacteria	Gammaproteo.	Alteromonadales	Alteromonadaceae	Glaciecola	*Glaciecola punicea*
**31**	Actinobacteria	Actinobacteria	Actinomycetales	Microbacteriaceae	Candidatus Aquiluna	*Candidatus Aquiluna rubra*
**34**	Proteobacteria	Betaproteo.	Burkholderiales	Comamonadaceae	RS62	uncl. RS62
**37**	Proteobacteria	Gammaproteo.	Thiohalorhabdales	uncl. Thiohalorhabdales	uncl. Thiohalorhabdales	uncl. Thiohalorhabdales
**41**	Bacteroidetes	Flavobacteriia	Flavobacteriales	Flavobacteriaceae	Maribacter	uncl. Maribacter
**44**	Bacteroidetes	BME43	uncl. BME43	uncl. BME43	uncl. BME43	uncl. BME43
**45**	Proteobacteria	Betaproteo.	Methylophilales	Methylophilaceae	uncl. Methylophilaceae	uncl. Methylophilaceae
**46**	Bacteroidetes	Flavobacteriia	Flavobacteriales	NS9	uncl. NS9	uncl. NS9
**49**	Actinobacteria	Acidimicrobiia	Acidimicrobiales	uncl. Acidimicrobiales	uncl. Acidimicrobiales	uncl. Acidimicrobiales
**51**	Proteobacteria	Alphaproteo.	Rhodobacterales	Hyphomonadaceae	uncl. Hyphomonadaceae	uncl. Hyphomonadaceae
**52**	Proteobacteria	Betaproteo.	uncl. Betaproteo.	uncl. Betaproteo.	uncl. Betaproteo.	uncl. Betaproteo.
**59**	Bacteroidetes	Flavobacteriia	Flavobacteriales	Flavobacteriaceae	uncl. Flavobacteriaceae	uncl. Flavobacteriaceae
**61**	Bacteroidetes	Flavobacteriia	Flavobacteriales	Flavobacteriaceae	uncl. Flavobacteriaceae	uncl. Flavobacteriaceae
**63**	Proteobacteria	Alphaproteo.	uncl. Alphaproteo.	uncl. Alphaproteo.	uncl. Alphaproteo.	uncl. Alphaproteo.
**68**	uncl. Bacteria	uncl. Bacteria	uncl. Bacteria	uncl. Bacteria	uncl. Bacteria	uncl. Bacteria
**70**	Actinobacteria	Acidimicrobiia	Acidimicrobiales	C111	uncl. C111	uncl. C111
**87**	Bacteroidetes	[Saprospirae]	[Saprospirales]	Saprospiraceae	Rubidimonas	*Rubidimonas crustatorum*
