# Supplementary material for: Warming, but Not Acidification, Restructures Epibacterial Communities of the Baltic Macroalga Fucus vesiculosus With Seasonal Variability
Source: Front Microbiol. 2020 Jun 26;11:1471. doi: 10.3389/fmicb.2020.01471 (PMC7333354; doi:10.3389/fmicb.2020.01471)
Supplement: Supplementary file 10 [file Data_Sheet_10.PDF]

Tab. S3 Week-wise and Temp evaluation. Results of the week- and temperature level-wise evaluation of significant (interaction) terms based on Redundancy Analysis (RDA) submodels per season. F-, *p*- and *q*-values (Benjamini-Hochberg-adjusted) as well as adjusted R^2 values are given for each evaluated term. dfn, numerator degrees of freedom; dfd, denominator degrees of freedom; Temp, Temperature; CE, Core Experiment; ∪, unification.

Season	Evaluated term	dfn	dfd	$F_{(dfn,dfd)}$	*p*	*q*	adjusted R^2
**Spring**	**Temp within constant Type and Week**						
	Biofilm_Fucus.Temp.Week_CE1_00	1	8	0.84	0.710	0.710	-0.02
	Biofilm_Fucus.Temp.Week_CE1_04	1	10	1.75	0.026	0.035	0.06
	Biofilm_Fucus.Temp.Week_CE1_08	1	10	2.18	0.005	0.014	0.10
	Biofilm_Fucus.Temp.Week_CE1_11	1	10	1.89	0.007	0.014	0.07
	Water.Temp.Week_CE1_00	1	10	0.85	0.740	0.740	-0.01
	Water.Temp.Week_CE1_04	1	10	2.84	0.007	0.009	0.14
	Water.Temp.Week_CE1_08	1	10	3.44	0.006	0.009	0.18
	Water.Temp.Week_CE1_11	1	10	3.04	0.007	0.009	0.16
**Summer**	**Temp within constant Type and Week**						
	Biofilm_Fucus.Temp.Week_CE2_00	1	10	1.68	0.031	0.031	0.06
	Biofilm_Fucus.Temp.Week_CE2_04	1	10	2.79	0.003	0.005	0.14
	Biofilm_Fucus.Temp.Week_CE2_08	1	10	4.15	0.001	0.003	0.22
	Water.Temp.Week_CE2_00	1	10	0.42	0.919	0.919	-0.06
	Water.Temp.Week_CE2_04	1	10	2.00	0.005	0.008	0.08
	Water.Temp.Week_CE2_08	1	10	2.97	0.002	0.006	0.15
**Autumn**	**Week within constant Type**						
	**Biofilm_Fucus:**						
	Week_CE3_00 - Week_CE3_10	1	22	6.53	<0.001	<0.001	0.19
	Week_CE3_00 - Week_CE3_04	1	22	5.65	<0.001	<0.001	0.17
	Week_CE3_04 - Week_CE3_10	1	22	3.13	<0.001	<0.001	0.08
	**Water:**						
	Week_CE3_00 - Week_CE3_10	1	22	7.38	<0.001	<0.001	0.22
	Week_CE3_00 - Week_CE3_04	1	22	4.72	<0.001	<0.001	0.14
	Week_CE3_04 - Week_CE3_10	1	22	4.56	<0.001	<0.001	0.13
**Winter**	**Temp, controlled for Type and Week**						
	Temp	1	85	2.17	0.001	0.001	0.01
	**Week within constant Type, controlled for Temp**						
	**Biofilm_Fucus:**						
	Week_CE4_00 - (Week_CE4_08 ∪ Week_CE4_11)	1	32	6.53	0.001	0.003	0.14
	Week_CE4_04 - (Week_CE4_08 ∪ Week_CE4_11)	1	32	3.30	0.001	0.003	0.06
	Week_CE4_00 - Week_CE4_04	1	21	3.06	0.001	0.003	0.09
	**Water:**						
	Week_CE4_00 - Week_CE4_08	1	21	7.48	0.001	0.006	0.23
	Week_CE4_00 - Week_CE4_11	1	21	7.24	0.001	0.006	0.22
	Week_CE4_08 - Week_CE4_11	1	21	6.53	0.001	0.006	0.19
	Week_CE4_04 - Week_CE4_11	1	20	5.24	0.001	0.006	0.16
	Week_CE4_04 - Week_CE4_08	1	20	5.16	0.001	0.006	0.16
	Week_CE4_00 - Week_CE4_04	1	20	2.65	0.001	0.006	0.07
