# Supplementary material for: Warming, but Not Acidification, Restructures Epibacterial Communities of the Baltic Macroalga Fucus vesiculosus With Seasonal Variability
Source: Front Microbiol. 2020 Jun 26;11:1471. doi: 10.3389/fmicb.2020.01471 (PMC7333354; doi:10.3389/fmicb.2020.01471)
Supplement: Supplementary file 11 [file Data_Sheet_11.PDF]

Tab. S4 Taxonomy and relative abundances of iOTUs for ambient temperature. Only iOTUs for ambient temperature with taxonomic information at least at genus level were listed. Mean relative abundances in ‰ of each iOTU for increased (‰ +T) or ambient (‰ aT) temperature level. The differences in relative abundances (Δ‰ = ‰ +T - ‰ aT) underline the negative (-) impact of the applied temperature treatment on these iOTUs compared to ambient conditions. The selected iOTUs are alphabetically sorted by class within season/week/type.

Season	Week	Type	OTU	Kingdom	Phylum	Class	Order	Family	Genus	Species	‰ +T	‰ aT	Δ‰
Spring	4	Fucus	245	Bacteria	Bacteroidetes	[Saprospirae]	[Saprospirales]	Saprospiraceae	Lewinella	unclassified	0.73	4.24	-3.51
Spring	4	Fucus	9	Bacteria	Bacteroidetes	Flavobacteriia	Flavobacteriales	Flavobacteriaceae	Flavobacterium	unclassified	0.00	1.34	-1.34
Spring	4	Fucus	838	Bacteria	Verrucomicrobia	Verrucomicrobiae	Verrucomicrobiales	Verrucomicrobiaceae	Luteolibacter	unclassified	0.65	2.78	-2.13
Spring	8	Fucus	864	Bacteria	Proteobacteria	Alphaproteobacteria	Rickettsiales	Rickettsiaceae	Rickettsia	unclassified	0.24	7.69	-7.45
Spring	8	Fucus	30	Bacteria	Proteobacteria	Gammaproteobacteria	Alteromonadales	Alteromonadaceae	Glaciecola	*Glaciecola punicea*	6.19	56.38	-50.19
Spring	11	Fucus	17	Bacteria	Proteobacteria	Alphaproteobacteria	Sphingomonadales	Erythrobacteraceae	Erythrobacter	unclassified	15.25	37.87	-22.62
Spring	11	Fucus	89	Bacteria	Bacteroidetes	Flavobacteriia	Flavobacteriales	Flavobacteriaceae	Psychroserpens	*Psychroserpens mesophilus*	0.01	2.57	-2.56
Spring	11	Fucus	30	Bacteria	Proteobacteria	Gammaproteobacteria	Alteromonadales	Alteromonadaceae	Glaciecola	*Glaciecola punicea*	1.23	17.75	-16.52
Spring	8	Water	1	Bacteria	Proteobacteria	Alphaproteobacteria	Rickettsiales	Pelagibacteraceae	Pelagibacter	*Pelagibacter ubique*	290.00	480.14	-190.14
Spring	11	Water	31	Bacteria	Actinobacteria	Actinobacteria	Actinomycetales	Microbacteriaceae	Candidatus Aquiluna	*Candidatus Aquiluna rubra*	0.31	27.76	-27.45
Spring	11	Water	9	Bacteria	Bacteroidetes	Flavobacteriia	Flavobacteriales	Flavobacteriaceae	Flavobacterium	unclassified	12.22	44.88	-32.66
Spring	11	Water	43	Bacteria	Bacteroidetes	Flavobacteriia	Flavobacteriales	Flavobacteriaceae	Sediminicola	unclassified	0.09	61.96	-61.87
Summer	4	Fucus	420	Bacteria	Proteobacteria	Alphaproteobacteria	Rhodobacterales	Hyphomonadaceae	Hirschia	*Hirschia baltica*	0.00	7.87	-7.87
Summer	4	Fucus	17	Bacteria	Proteobacteria	Alphaproteobacteria	Sphingomonadales	Erythrobacteraceae	Erythrobacter	unclassified	13.94	39.48	-25.54
Summer	4	Fucus	132	Bacteria	Bacteroidetes	Cytophagia	Cytophagales	Flammeovirgaceae	Roseivirga	unclassified	0.00	2.41	-2.41
Summer	4	Fucus	11	Bacteria	Proteobacteria	Gammaproteobacteria	Alteromonadales	Alteromonadaceae	Glaciecola	unclassified	0.32	15.48	-15.16
Summer	4	Fucus	72	Bacteria	Cyanobacteria	Nostocophycideae	Nostocales	Nostocaceae	Anabaena	*Anabaena cylindrica*	0.12	10.00	-9.88
Summer	8	Fucus	87	Bacteria	Bacteroidetes	[Saprospirae]	[Saprospirales]	Saprospiraceae	Rubidimonas	*Rubidimonas crustatorum*	0.00	9.66	-9.66
Summer	8	Fucus	829	Bacteria	Bacteroidetes	Flavobacteriia	Flavobacteriales	Cryomorphaceae	Crocinitomix	unclassified	0.00	1.48	-1.48
Summer	4	Water	9	Bacteria	Bacteroidetes	Flavobacteriia	Flavobacteriales	Flavobacteriaceae	Flavobacterium	unclassified	5.25	25.19	-19.94
Summer	4	Water	90	Bacteria	Proteobacteria	Gammaproteobacteria	Alteromonadales	Alteromonadaceae	Glaciecola	unclassified	0.22	20.40	-20.18
Summer	4	Water	571	Bacteria	Proteobacteria	Gammaproteobacteria	Alteromonadales	Alteromonadaceae	HTCC2207	unclassified	0.15	1.03	-0.88
Summer	4	Water	1048	Bacteria	Proteobacteria	Gammaproteobacteria	Pseudomonadales	Pseudomonadaceae	Pseudomonas	unclassified	0.00	0.35	-0.35
Summer	4	Water	924	Bacteria	Verrucomicrobia	Opitutae	Puniceicoccales	Puniceicoccaceae	Coraliomargarita	unclassified	0.00	0.34	-0.34
Summer	4	Water	541	Bacteria	Verrucomicrobia	Verrucomicrobiae	Verrucomicrobiales	Verrucomicrobiaceae	Persicirhabdus	unclassified	0.03	0.52	-0.49
Summer	8	Water	94	Bacteria	Actinobacteria	Actinobacteria	Actinomycetales	Microbacteriaceae	Microbacterium	unclassified	0.02	2.41	-2.39
Summer	8	Water	214	Bacteria	Actinobacteria	Actinobacteria	Actinomycetales	Microbacteriaceae	Yonghaparkia	unclassified	1.32	4.21	-2.89
Summer	8	Water	3	Bacteria	Proteobacteria	Alphaproteobacteria	Rhodobacterales	Rhodobacteraceae	Octadecabacter	unclassified	55.73	112.81	-57.08
Summer	8	Water	27	Bacteria	Proteobacteria	Gammaproteobacteria	Alteromonadales	Alteromonadaceae	Glaciecola	*Glaciecola mesophila*	0.02	2.39	-2.37
Summer	8	Water	5	Bacteria	Proteobacteria	Gammaproteobacteria	Oceanospirillales	Halomonadaceae	Candidatus Portiera	unclassified	9.11	25.58	-16.47
Winter			220	Bacteria	Bacteroidetes	[Saprospirae]	[Saprospirales]	Saprospiraceae	Lewinella	unclassified	1.73	2.98	-1.25
Winter			1436	Bacteria	Bacteroidetes	[Saprospirae]	[Saprospirales]	Saprospiraceae	Lewinella	unclassified	0.02	0.62	-0.60
Winter			398	Bacteria	Bacteroidetes	Flavobacteriia	Flavobacteriales	Cryomorphaceae	Fluviicola	unclassified	0.06	0.64	-0.58
Winter			2153	Bacteria	Bacteroidetes	Flavobacteriia	Flavobacteriales	Cryomorphaceae	Fluviicola	unclassified	0.21	0.56	-0.35
Winter			41	Bacteria	Bacteroidetes	Flavobacteriia	Flavobacteriales	Flavobacteriaceae	Maribacter	unclassified	2.77	5.77	-3.00
Winter			905	Bacteria	Bacteroidetes	Flavobacteriia	Flavobacteriales	Flavobacteriaceae	Polaribacter	unclassified	0.08	0.40	-0.32
Winter			1375	Bacteria	Proteobacteria	Gammaproteobacteria	Alteromonadales	Alteromonadaceae	Glaciecola	*Glaciecola psychrophila*	0.05	0.74	-0.69
Winter			882	Bacteria	Proteobacteria	Gammaproteobacteria	Alteromonadales	Alteromonadaceae	Glaciecola	unclassified	0.30	1.14	-0.84
Winter			541	Bacteria	Verrucomicrobia	Verrucomicrobiae	Verrucomicrobiales	Verrucomicrobiaceae	Persicirhabdus	unclassified	0.26	0.67	-0.41
Winter			611	Bacteria	Verrucomicrobia	Verrucomicrobiae	Verrucomicrobiales	Verrucomicrobiaceae	Roseibacillus	unclassified	0.44	1.18	-0.74
