# Supplementary material for: Warming, but Not Acidification, Restructures Epibacterial Communities of the Baltic Macroalga Fucus vesiculosus With Seasonal Variability
Source: Front Microbiol. 2020 Jun 26;11:1471. doi: 10.3389/fmicb.2020.01471 (PMC7333354; doi:10.3389/fmicb.2020.01471)
Supplement: Supplementary file 12 [file Data_Sheet_12.PDF]

Tab. S5 Taxonomy and relative abundances of iOTUs for increased temperature. Only iOTUs for aT with taxonomic information at least at genus level were listed. Mean relative abundances in ‰ of each iOTU for increased (‰ +T) or ambient (‰ aT) temperature level. The differences in relative abundances (Δ‰ = ‰ +T - ‰ aT) underline the positive (+) impact of the applied temperature treatment on these iOTUs compared to ambient conditions. The selected iOTUs are alphabetically sorted by class within season/week/type.

Season	Week	Type	OTU	Kingdom	Phylum	Class	Order	Family	Genus	Species	‰ +T	‰ aT	Δ‰
Spring	8	Water	191	Bacteria	Actinobacteria	Actinobacteria	Actinomycetales	Mycobacteriaceae	Mycobacterium	unclassified	2.18	0.03	+2.15
Spring	8	Water	73	Bacteria	Proteobacteria	Epsilonproteobacteria	Campylobacterales	Campylobacteraceae	Arcobacter	unclassified	8.66	0.06	+8.60
Spring	8	Water	1204	Bacteria	Proteobacteria	Epsilonproteobacteria	Campylobacterales	Campylobacteraceae	Arcobacter	unclassified	1.18	0.00	+1.18
Spring	8	Water	145	Bacteria	Bacteroidetes	Flavobacteriia	Flavobacteriales	Cryomorphaceae	Owenweeksia	unclassified	2.79	0.11	+2.68
Summer	4	Fucus	484	Bacteria	Bacteroidetes	Cytophagia	Cytophagales	Flammeovirgaceae	Roseivirga	unclassified	5.24	0.21	+5.03
Summer	4	Fucus	301	Bacteria	Proteobacteria	Epsilonproteobacteria	Campylobacterales	Campylobacteraceae	Arcobacter	unclassified	31.55	0.00	+31.55
Summer	8	Fucus	1793	Bacteria	Proteobacteria	Deltaproteobacteria	Myxococcales	Nannocystaceae	Plesiocystis	unclassified	3.14	0.00	+3.14
Summer	8	Fucus	1089	Bacteria	Bacteroidetes	Flavobacteriia	Flavobacteriales	Cryomorphaceae	Crocinitomix	unclassified	2.70	0.00	+2.70
Summer	8	Fucus	850	Bacteria	Bacteroidetes	Flavobacteriia	Flavobacteriales	Flavobacteriaceae	Maribacter	unclassified	1.73	0.05	+1.68
Summer	8	Fucus	246	Bacteria	Bacteroidetes	Flavobacteriia	Flavobacteriales	Flavobacteriaceae	Winogradskyella	*Winogradskyella poriferorum*	3.24	0.03	+3.21
Summer	8	Fucus	181	Bacteria	Cyanobacteria	Nostocophycideae	Stigonematales	Rivulariaceae	Rivularia	unclassified	10.01	1.22	+8.79
Summer	8	Fucus	528	Bacteria	Cyanobacteria	Oscillatoriophycideae	Oscillatoriales	Phormidiaceae	Phormidium	unclassified	2.18	0.00	+2.18
Summer	4	Water	301	Bacteria	Proteobacteria	Epsilonproteobacteria	Campylobacterales	Campylobacteraceae	Arcobacter	unclassified	0.95	0.00	+0.95
Summer	4	Water	1647	Bacteria	Proteobacteria	Gammaproteobacteria	Alteromonadales	Alteromonadaceae	BD2-13	unclassified	0.61	0.02	+0.59
Summer	8	Water	1115	Bacteria	Proteobacteria	Alphaproteobacteria	Kiloniellales	Kiloniellaceae	Thalassospira	unclassified	2.22	0.01	+2.21
Summer	8	Water	347	Bacteria	Bacteroidetes	Flavobacteriia	Flavobacteriales	Flavobacteriaceae	Sediminicola	unclassified	8.38	0.04	+8.34
Summer	8	Water	731	Bacteria	Proteobacteria	Gammaproteobacteria	Alteromonadales	Alteromonadaceae	Glaciecola	unclassified	3.90	0.00	+3.90
Summer	8	Water	24	Bacteria	Tenericutes	Mollicutes	Acholeplasmatales	Acholeplasmataceae	Acholeplasma	unclassified	29.82	4.93	+24.89
Winter			1843	Bacteria	Proteobacteria	Alphaproteobacteria	Rhizobiales	Hyphomicrobiaceae	Devosia	unclassified	0.64	0.09	+0.55
Winter			88	Bacteria	Proteobacteria	Alphaproteobacteria	Rhodobacterales	Rhodobacteraceae	Octadecabacter	*Octadecabacter antarcticus*	3.31	1.52	+1.79
Winter			17	Bacteria	Proteobacteria	Alphaproteobacteria	Sphingomonadales	Erythrobacteraceae	Erythrobacter	unclassified	13.21	8.64	+4.57
Winter			728	Bacteria	Bacteroidetes	Cytophagia	Cytophagales	Cytophagaceae	Leadbetterella	unclassified	1.46	0.11	+1.35
Winter			547	Bacteria	Bacteroidetes	Cytophagia	Cytophagales	Flammeovirgaceae	Reichenbachiella	unclassified	0.64	0.14	+0.50
Winter			551	Bacteria	Bacteroidetes	Cytophagia	Cytophagales	Flammeovirgaceae	Reichenbachiella	unclassified	0.44	0.01	+0.43
Winter			199	Bacteria	Proteobacteria	Deltaproteobacteria	Myxococcales	Nannocystaceae	Plesiocystis	unclassified	1.79	0.42	+1.37
Winter			1196	Bacteria	Proteobacteria	Deltaproteobacteria	Myxococcales	Nannocystaceae	Plesiocystis	unclassified	0.56	0.21	+0.35
Winter			1120	Bacteria	Bacteroidetes	Flavobacteriia	Flavobacteriales	Cryomorphaceae	Crocinitomix	unclassified	0.34	0.02	+0.32
Winter			603	Bacteria	Bacteroidetes	Flavobacteriia	Flavobacteriales	Cryomorphaceae	Fluviicola	unclassified	1.83	0.08	+1.75
Winter			113	Bacteria	Bacteroidetes	Flavobacteriia	Flavobacteriales	Flavobacteriaceae	Formosa	unclassified	3.05	1.66	+1.39
Winter			492	Bacteria	Bacteroidetes	Flavobacteriia	Flavobacteriales	Flavobacteriaceae	Polaribacter	unclassified	3.10	0.15	+2.95
Winter			870	Bacteria	Bacteroidetes	Flavobacteriia	Flavobacteriales	Flavobacteriaceae	Polaribacter	unclassified	0.77	0.06	+0.71
Winter			350	Bacteria	Proteobacteria	Gammaproteobacteria	Alteromonadales	Alteromonadaceae	Candidatus Endobugula	unclassified	2.55	0.08	+2.47
Winter			27	Bacteria	Proteobacteria	Gammaproteobacteria	Alteromonadales	Alteromonadaceae	Glaciecola	*Glaciecola mesophila*	15.80	4.91	+10.89
Winter			1548	Bacteria	Proteobacteria	Gammaproteobacteria	Alteromonadales	Alteromonadaceae	Umboniibacter	unclassified	1.16	0.03	+1.13
